# Fertility-Sparing Management of Atypical Hyperplasia and Endometrial Cancer from the Perspective of Molecular and Hormonal Profiles: A Meta-Analysis-Driven Framework

**DOI:** 10.3390/cancers18152490

**Published:** 2026-08-04

**Authors:** Myriam Jerbaka, Radwa Hablase, Alexander Shushkevich, Martin Koskas, Christopher El Hadi, Nadine El Kassis, Wissam Arab, David Atallah, Jayanta Chatterjee

**Affiliations:** 1 Department of Gynecological Surgery, Hotel-Dieu de France University Hospital, Saint-Joseph University, Beirut 16-6830, Lebanon; myriam.jerbaka@net.usj.edu.lb (M.J.); wissam.arab@mft.nhs.uk (W.A.); 2 Division of Gynecologic Oncology, Bichat University Hospital, Paris Cité University, 75018 Paris, France; martin.koskas@aphp.fr; 3 Faculty of Health, Sorbonne University, 75013 Paris, France; 4 Academic Department of Gynaeoncology, Royal Surrey NHS Foundation Trust, Guildford GU2 7XX, UK; radwa_noreen@hotmail.co.uk (R.H.); jayanta.chatterjee1@nhs.net (J.C.); 5 Department of Gynecology and Gynecologic Oncology, Evangelische Kliniken Essen Mitte, 45136 Essen, Germany; a.shushkevich.private@gmail.com; 6 Faculty of Medicine, Lebanese American University, Beirut 13-5053, Lebanon; chriselhadi@gmail.com; 7 Women’s Cancer Institute, Beirut 16-6830, Lebanon; 8 Saint Mary’s Hospital, Manchester University NHS Foundation Trust, Manchester M13 9WL, UK

**Keywords:** fertility-sparing, conservative management, molecular classification, endometrial cancer, atypical hyperplasia

## Abstract

**Background/Objectives**: The prognostic and predictive values of biomarkers in fertility-sparing management of atypical hyperplasia (AH) and endometrial cancer (EC) remain ill-defined. We aimed to identify the impact of tumour profiles on oncologic and reproductive outcomes to inform clinical decision-making. **Methods**: We conducted a systematic review and meta-analysis by searching MEDLINE, PubMed, Embase, Cochrane Library, Scopus, Google Scholar, and ClinicalTrials.gov, up to July 2026. We intended to include comparative studies or clinical trials, in English or French, assessing outcomes according to molecular or hormonal profiles in reproductive-aged women diagnosed with AH or EC. The primary outcome was the best overall complete remission (CR). Pooled odds ratios (ORs) were calculated using a random-effects model with logit transformation and restricted maximum likelihood estimation. Risk of bias was assessed using the Newcastle–Ottawa scale (NOS). The study protocol was registered in PROSPERO (CRD42025632885). **Results**: Eighteen retrospective studies comprising 965 patients were included. No specific molecular profile (NSMP) tumours demonstrated significantly higher odds of CR (OR 2.04, 95% CI 1.33–3.11). p53-abnormal (p53abn) and deficient mismatch repair (dMMR) tumours were significantly less likely to achieve CR compared to NSMP (OR 3.87, 95% CI 1.80–8.29 and OR 2.48, 95% CI 1.44–4.29, respectively). POLE-mutated (POLEmut) tumours showed CR comparable to NSMP (OR 1.39, 95% CI 0.60–3.24). Progesterone receptor (PR) positivity was strongly associated with CR (OR 7.73, 95% CI 2.77–21.63). **Conclusions**: NSMP and PR-positivity represented a favourable prognosis and potential prediction of CR. POLEmut tumours demonstrated CR rates comparable to NSMP, whereas p53abn and dMMR demonstrated unfavourable outcomes. These findings support a biologically tailored approach to patient selection for fertility-sparing management.

## Simple Summary

Molecular classification has become central in decision-making for fertility-sparing management of atypical hyperplasia (AH) and endometrial cancer (EC), although its prognostic and predictive values remain incompletely defined. We performed a systematic review and meta-analysis to determine the association between tumour profiles and oncologic outcomes in reproductive-aged women undergoing conservative treatment. Eighteen studies were included after searches of databases up to July 2026. p53-abnormal (p53abn) and deficient mismatch repair (dMMR) tumours were significantly associated with lower complete remission (CR) rates compared with other subgroups. In contrast, no specific molecular profile (NSMP) tumours were associated with higher odds of CR. POLE-mutated (POLEmut) tumours showed CR rates comparable to NSMP without significant differences. Progesterone receptor (PR) positivity was strongly associated with CR. These findings support biologically stratified selection for fertility-sparing management for EC and AH.

## 1. Introduction

Endometrial cancer is the most prevalent malignancy of the female reproductive system. According to Cancer Research United Kingdom, 1 in every 39 women will be diagnosed with uterine cancer in their lifetime [1]. In the United States and Europe, age-specific incidence rates rise steeply from the age of 45 years onwards, with 7.1% of the cases occurring in women younger than this age [2,3]. In this population of reproductive-aged women, fertility-sparing management is an important consideration, particularly for nulliparous patients, who constitute many of the endometrial cancer cases within this age group [4,5,6,7]. The recommended management for early-stage endometrial cancer is total hysterectomy with bilateral salpingo-oophorectomy (+/− nodal assessment). However, if childbearing is desired, fertility-sparing management could have good outcomes when the tumour is confined to the endometrium without myometrial invasion (FIGO stage IA1) [7,8].

Endometrial hyperplasia is a proliferation of the endometrial lining, with an increase in gland-to-stroma ratio compared to normal proliferative endometrium. When cytological atypia occurs, surgical management is recommended. This includes total hysterectomy with bilateral salpingectomy (+/− oophorectomy), due to the risk of underlying unsampled malignancy, as well as the risk of progression to cancer [9]. In pre-menopausal women, the reported prevalence of atypical hyperplasia ranges from 3.4 to 7.9%, hence the importance of proposing fertility-sparing management in this age group [10].

Oral or local progestins constitute the main treatment modality [7]. Hysteroscopic resection has also been combined with progesterone to improve outcomes in several studies [11,12,13,14]. Following complete remission (CR) and until parenthood is completed, close surveillance with endometrial biopsy every three to six months is recommended [9]. However, a significant number of patients either do not respond to conservative treatment or relapse after a remission, increasing the risk of developing an invasive disease [15]. As a result, prognostic indicators that could forecast the likelihood of response and relapse during fertility-sparing treatment have recently attracted more attention. Identifying favourable prognostic factors could improve the safety of fertility-sparing management by helping guide decisions regarding the optimal timing of pregnancy and the duration of conservative treatment before attempting conception.

Molecular classification is now one of the most important and the most biologically informative prognostic factors in endometrial cancer. According to The Cancer Genome Atlas (TCGA), endometrial carcinoma is classified into four molecular subtypes: deficient mismatch repair (dMMR), polymerase epsilon mutated (POLEmut), p53-abnormal (p53abn), and non-specific molecular profile (NSMP). A tumour’s molecular profile has become a promising tool in tailoring radical management plans preoperatively by classifying tumours into different prognostic risk groups (low, intermediate, high-intermediate, or high-risk group) [10]. Molecular signature is also a predictor of nodal involvement, response to adjuvant therapy, and risk of relapse [16]. Progesterone receptor (PR)-positive tumours have also shown better responsiveness to hormonal treatment [17].

Thus, we aimed to conduct a systematic review and meta-analysis on the available evidence regarding the prognostic and predictive values of molecular and hormonal biomarkers in reproductive-aged women undergoing fertility-sparing management for atypical hyperplasia or early-stage endometrial cancer. Specifically, we sought to estimate their prognostic role and their ability to predict CR to hormonal therapy and to stratify the risk of disease recurrence or progression, and the CR incidence over time.

## 2. Materials and Methods

### 2.1. Study Design and Objectives

We conducted a systematic review and meta-analysis in accordance with the *Cochrane Handbook for Systematic Reviews of Interventions* and followed the Preferred Reporting Items for Systematic Reviews and Meta-Analyses 2020 (PRISMA 2020) guidelines [18], aiming to evaluate the prognostic role of molecular subtypes and hormonal receptors after fertility-sparing management in patients with atypical hyperplasia or early-stage endometrial carcinoma.

### 2.2. Eligibility Criteria

Studies were eligible if they (1) enrolled at least 10 reproductive-aged patients undergoing fertility-sparing treatment; (2) reported oncologic outcomes with or without reproductive outcomes stratified by endometrial molecular classification; and (3) were randomized controlled trials, comparative cohort studies, or case–control studies published in English or French. Exclusion criteria were: non-English/French, non-clinical, <10 patients, reviews, conference abstracts, case reports, letters, non-comparative designs, or had non-extractable data.

### 2.3. Data Sources and Search Strategy

A comprehensive search of MEDLINE, PubMed, Embase, Cochrane Library, and ClinicalTrials.gov identified relevant studies published up to July 2026. Search terms combined MeSH and keywords related to molecular prognostic factors and fertility-sparing management, including: “biomolecular prognostic factors”, “genetic factors”, “estrogen receptor”, “progesterone receptor”, “molecular classification”, “endometrial neoplasms”, “hyperplasia”, “gonadotropin-releasing hormone”, “hysteroscopy”, “medroxyprogesterone acetate”, “megestrol acetate”, “progestins”, “levonorgestrel”, “intrauterine devices”, “fertility preservation”, “conservative treatment”, “hormonal therapy”, and “fertility-sparing”. The detailed search strategy is provided in the Supplementary Materials. Reference lists of all included articles were manually screened using Scopus (Elsevier) and Google Scholar to identify additional eligible studies.

All records were imported into the Rayyan application (Rayyan Systems Inc., version 1.7.7., Cambridge, MA, USA; web version, accessed 18 November 2025) for deduplication, blinded title/abstract and full-text screening, and tracking of study selection [19]. The review protocol was prospectively registered in PROSPERO (CRD42025632885) [20].

### 2.4. Outcomes and Definitions

The primary outcome was the odds ratio (OR) for best overall CR according to molecular/hormonal status. Secondary outcomes included: pooled rates of progression, pooled median time to remission (TTR), pooled recurrence rate after CR, pooled median time to recurrence, and reproductive outcomes (pregnancy and live birth rates) when reported.

For tumours with multiple molecular classifiers, signatures were assigned as follows: POLEmut-dMMR-p53abn or POLEmut-p53abn were classified as POLEmut; dMMR-p53abn lesions were classified as dMMR [21]. When germline testing confirmed Lynch syndrome, cases were classified as dMMR. In tumours with concurrent POLEmut and Lynch syndrome, assignment to the dMMR group was retained for analytical consistency. These cases were identified through germline testing and DNA-based analyses rather than by tumour immunohistochemistry or tumour next-generation sequencing. The optimal classification of POLEmut dMMR tumours remains an area of ongoing investigation [22]. On the other hand, pMMR, p53 wild-type, and POLE wild-type tumours correspond to the same group of patients, defining the NSMP category. These tumours showed no other molecular abnormalities that would allow assignment to another molecular subgroup, thereby avoiding misclassification.

### 2.5. Data Extraction and Quality Assessment

Three authors (M.J., R.H. and A.S.) reviewed the articles blindly and identified duplicates with the Rayyan application [19]. The quality of the observational studies was assessed using the Newcastle–Ottawa Scale (NOS) [23]. Disagreement was resolved by review of the study. A sensitivity analysis was done after exclusion to assess any duplication in populations when the studies were published by the same authors [24].

From each study, we obtained the first author’s name, publication year, country, study design, number of patients, demographic characteristics (age, body mass index (BMI)), histological diagnosis, pathology review, treatment protocols, all available biomarkers, CR at reported timepoint, best overall CR, time to CR, progression rate, recurrence after CR, and time to recurrence. The data of included studies were extracted into an Excel sheet.

### 2.6. Statistical Analysis

All analyses were conducted in R version 4.4.2 (31 October 2024) using the *meta* (version 7.0-0) and *metafor* (version 4.6-0) packages. All statistical tests were two-sided, with *p* < 0.05 considered statistically significant. Statistical heterogeneity was assessed using I^2^, τ^2^, and Cochran’s Q test.

For the primary comparison of marker-positive versus marker-negative groups, 2 × 2 contingency tables were constructed for each gene–study pair and synthesized using the Mantel–Haenszel method implemented in *metabin()*. Random-effects models were applied using the restricted maximum likelihood (REML) estimator for between-study variance (τ^2^). A continuity correction of 0.5 was applied to studies with zero cells. Effect estimates were pooled on the logarithmic scale and subsequently back-transformed for reporting as odds ratios (ORs) with corresponding confidence intervals.

Time to treatment response (TTR) was pooled using inverse variance weighting of study-level summary estimates (medians or means). When only medians with ranges or interquartile ranges were reported, means and standard deviations were estimated using the method proposed by Wan et al. [25].

Study-level recurrence proportions after CR were pooled using *metaprop* with logit-transformed proportions (proportion log odds, PLO) within a multilevel random-effects framework, accounting for study-level clustering. Pooled estimates were subsequently back-transformed to the proportion scale for interpretation. Meta-regression analyses were performed using *rma.mv*, with study author included as a random intercept, to explore potential moderators of treatment response, including publication year, gene, and molecular classification. The PLO effect size was used consistently for both marker-positive and marker-negative groups.

Pseudo-individual patient data (pseudo-IPD) were reconstructed from aggregate interval-based response data using established reconstruction methods to enable exploratory Kaplan–Meier estimation of time to CR [25,26,27]. Briefly, the full enrolled cohort (y_0_ = total enrolled patients) was considered at risk at time zero. Within each consecutive interval [t_i−1_, t_i_], newly observed CR events (x_i_ − x_i−1_) were assigned event times sampled from a uniform distribution between the interval boundaries. Patients who discontinued follow-up without achieving CR during interval i (y_i−1_ − y_i_) were censored at the midpoint of the corresponding interval, whereas patients remaining under observation at the final assessment without CR (y_last − x_last) were censored at t_last. A fixed random seed was used to ensure reproducibility, and internal consistency checks confirmed that the total number of reconstructed events and censoring observations equalled the reported enrolled cohort size for each study.

Because pseudo-IPD are derived from aggregate rather than individual-level observations, this reconstruction approach relies on assumptions that may introduce uncertainty and potential bias. In particular, the assumption of uniformly distributed CR events within reporting intervals and midpoint censoring may not fully reflect the true temporal distribution of remission events or losses to follow-up. In addition, reconstructed datasets cannot capture individual patient characteristics, baseline covariates, treatment heterogeneity, or the original censoring mechanisms. Therefore, the reconstructed Kaplan–Meier curves should be interpreted as descriptive approximations rather than exact estimates of patient-level time-to-event outcomes. These analyses were performed primarily to visualize the dynamics of CR achievement across molecular groups and were considered exploratory and hypothesis-generating. The primary statistical inferences were based on aggregate-level random-effects meta-analyses rather than the reconstructed survival curves.

Kaplan–Meier curves were presented as cumulative incidence functions (1 − S(t)), increasing from 0% CR at baseline, with Greenwood 95% confidence intervals. At each predefined time point, the proportion of patients achieving CR among the total number of evaluable living patients (including those with and without CR at the end of follow-up) was pooled within each molecular group using random-effects meta-analysis with REML estimation of between-study variance (*metafor* package). Proportions were logit-transformed before modelling and back-transformed for graphical presentation. A continuity correction of 10^−6^ was applied to account for proportions equal to zero or one. A matching tolerance of ±0.7 months was used to accommodate the annotation corrections described above. A time-zero anchor corresponding to 0% CR was added to each reconstructed curve. Consensus curves were generated separately for the three analytical frameworks and in a combined all-groups analysis, with 95% confidence interval ribbons and point sizes scaled according to the number of contributing studies (k) at each time point.

For gene–outcome combinations with at least three contributing studies, potential publication bias was assessed visually using funnel plots and statistically using Egger’s weighted regression test. A *p*-value < 0.10 was considered suggestive of small-study effects. Funnel plots are provided in the Supplementary Materials.

## 3. Results

**Figure 1:**
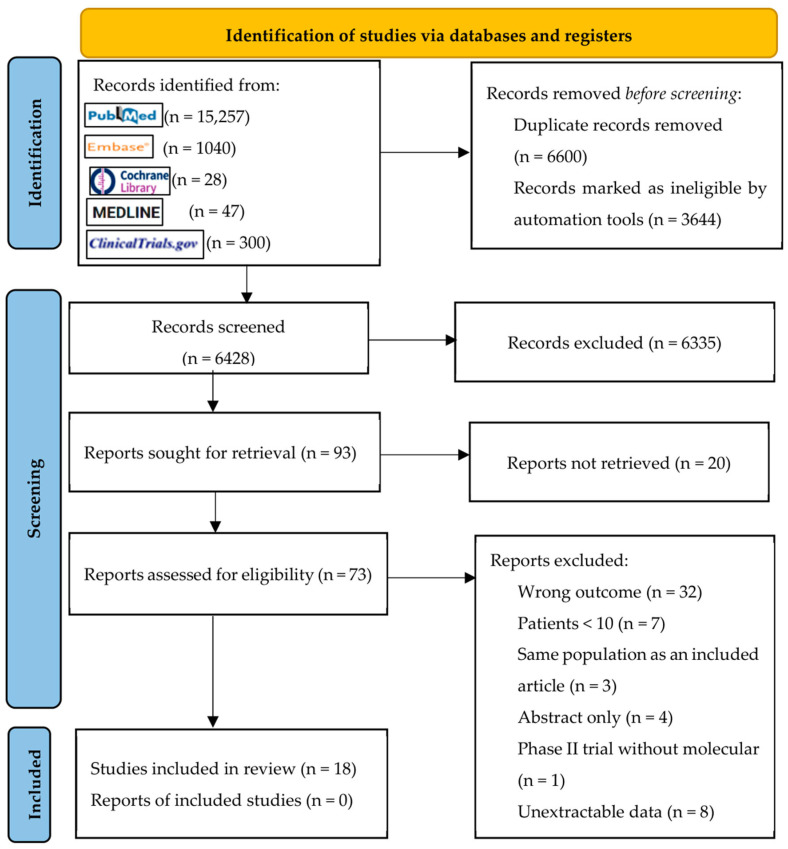
PRISMA 2020 flow diagram for new systematic reviews which included searches of databases, registers and other sources. Source: Page, M.J. et al. *BMJ* **2021**; *372*, n71. doi: 10.1136/bmj.n71 [18]. This work is licenced under CC BY 4.0. To view a copy of this licence, visit https://creativecommons.org/licenses/by/4.0/ (accessed on 30 December 2025).

The search identified 16,972 records; 10,072 were screened after deduplication, and 73 underwent full-text review. Overall, 18 retrospective cohorts were included (Asia n = 13, Europe n = 4, North America n = 1; Figure 1). Quality scores (Newcastle–Ottawa Scale) ranged from 5 to 9. A sensitivity analysis [24] was done, and we excluded two studies by Wang et al. [28,29], having the same setting, participants, and last author as Jiayi Wang et al. [30], and a phase II trial with high risk of bias [31]. The pooled cohort comprised 965 patients (mean age 32.6 years; mean BMI 26 kg/m^2^). Across the 18 studies, 15 distinct molecular markers were evaluable, generating 81 unique gene-study-outcome strata for the primary analysis, 140 strata for meta-regression of CR, and 88 strata for recurrence analysis (Table 1).

Molecular profiling was immunohistochemistry-based (IHC), with a subset employing next-generation sequencing (NGS). Most of the studies reported pathology review by two pathologists or more, underscoring variability in histopathologic interpretation and potential for misclassification-driven heterogeneity [30,32,33,34,35].

Fertility-sparing treatment consisted of oral progestin therapy, intrauterine progestin delivery, or combined approaches. The majority of studies used oral progestins or hormonal therapy alone, including progestin-based regimens with or without GnRH agonists [33,36,37,38,39]. LNG-IUD-based approaches were reported by De Vitis et al. and Lv et al., either alone or combined with systemic hormonal therapy [40,41]. Surgical approaches combined with hormonal therapy were reported by Falcone et al., Raffone et al., and Wang J. et al. [30,42,43]. Other studies evaluated multimodal or guideline-based hormonal approaches [31,44,45,46]. Overall, hormonal therapy remained the predominant fertility-sparing strategy, with hysteroscopic resection or other surgical approaches incorporated in selected cohorts [43,44,46,47].

**Table 1:** Summary of the included studies.

Author	Country	Year	NOS *	TCGA/ProMisE	Pathology Review	n	Follow-Up Median in Months (Range)	POLEmut	dMMR/MSI-H	p53abn	NSMP/CNL	Lynch Syndrome
Chung et al. [36]	Korea	2021	8	IHC/PCR|Sanger	Yes	57	38.4 (2.9–163.5)	2	9	1	45	NA
Dagher et al. [37]	USA	2023	7	IHC/NGS	Yes	20	48.0 (12–123)	1	3	NA	17	1
De Vitis et al. [40]	Italy	2024	8	IHC/NGS	Yes	33	100.5 (47–175)	3	3	2	25	3
Falcone et al. [42]	Italy	2019	8	IHC/NGS	Yes	15	106 (24–134)	2	6	0	7	2
Lv et al. [41]	China	2023	9	IHC/PCR|Sanger	Yes	93	24 (12–36)	6	15	5	67	NA
Van Gent et al. [35]	Netherlands	2016	8	IHC/PCR|Sanger	Yes	11	32 (19–128)	NA	NA	NA	NA	NA
Minaguchi et al. [34]	Japan	2006	7	IHC	NA	31	40.7 (2–109)	NA	NA	4	27	NA
Ota et al. [48]	Japan	2005	8	IHC	Yes	12	52.7 (13–154)	NA	NA	NA	NA	NA
Peng H. et al. [44]	China	2024	7	IHC	Yes	51	30 (6–80)	NA	11	6	34	4
Peng S. et al. [33]	China	2024	6	IHC/PCR|Sanger	Yes	80	22 (6.3–107.1)	5	7	4	64	NA
Raffone et al. [43]	Italy	2021	8	IHC	Yes	69	54.8	NA	6	NA	NA	NA
Ran et al. [45]	China	2022	8	IHC/PCR|Sanger	NA	13	54.7 (11–138)	NA	3	1	8	0
Utsunomiya et al. [32]	Japan	2002	7	IHC	Yes	16	7.3 (6–12)	NA	NA	NA	NA	NA
Wang J et al. [30]	China	2025	7	IHC/PCR|Sanger	Yes	103	53 (12–201)	3	26	7	67	NA
Wang Y et al. [38]	China	2023	6	NGS	NA	52	9 (4.5–13)	4	7	2	39	NA
Xu et al. [31]	China	2023	8	IHC/NGS	Yes	91	13.8 (4.41–24)	6	6	1	78	1
Xue et al. [46]	China	2023	8	IHC/NGS	Yes	135	13 (0.5–56)	1	14	3	117	2
Zhang et al. [39]	China	2023	8	NGS	Yes	83	37 (4–98)	NA	NA	1	45	NA

* NOS: Newcastle–Ottawa Scale, NA: Not applicable.

### 3.1. Primary Outcome: Complete Remission by Molecular Profile

Overall, 65% (72/116) dMMR tumours and 77% (488/631) pMMR tumours reached CR (OR 2.48, 95% CI 1.44–4.29). POLEmut and POLEwt tumours had a similar rate of CR (OR 1.39, 95% CI 0.60–3.24). Based on p53 status, CR rates were lower in p53abn tumours compared to p53wt tumours, with 56.7% (21/37) versus 76.8% (473/616), respectively (OR 3.87 95% CI 1.80–8.29).

**Figure 2:**
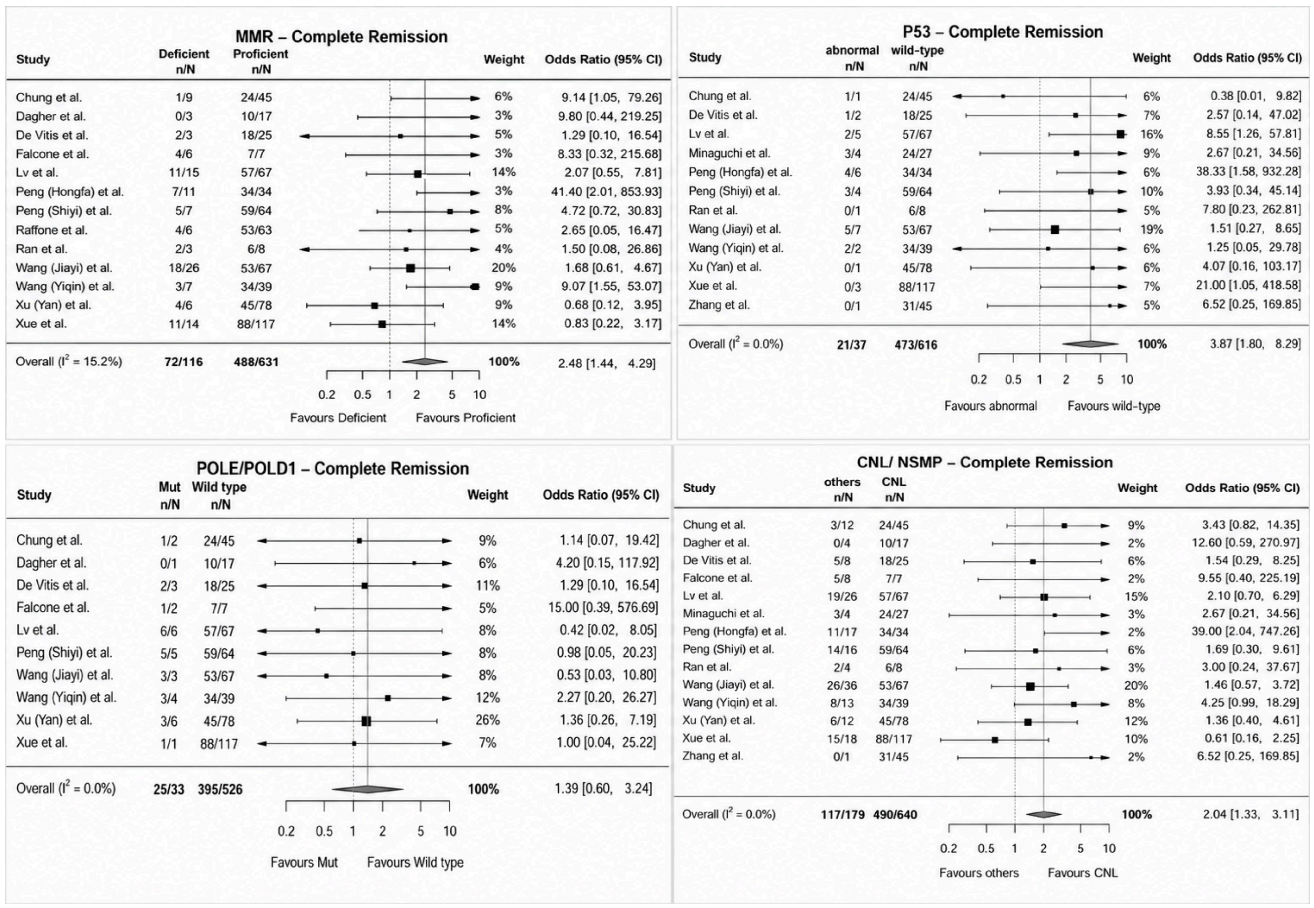
Forest plots for complete remission by molecular classification [30,31,33,34,36,37,38,39,40,41,42,43,44,45,46].

CR was frequently observed in NSMP, with 76.5% (490/640) responders compared to 65.4% (117/179) in the others group (OR 2.04, 95% CI 1.33–3.11). The results are represented in Figure 2 as forest plots.

Only three studies assessed response according to hormonal receptor (HR) status. Responders included 80% (140/175) PR-positive tumours and 42% (8/19) PR-negative (OR 7.73, 95% CI 2.77–21.63). As for ER, 78.4% (116/148) ER-positive tumours and 73.9% (17/23) ER-negative tumours reached CR; a small difference without statistical significance (OR 1.43, 95% CI 0.52–3.93).

Among somatic mutations, 40% (6/15) KRASmut and 59.3% (51/86) KRASwt responded (OR 1.8, 95% CI 0.34–9.50); the same is observed for PIK3CA, with 52.6% (20/38) reaching CR (OR 0.45, 95% CI 0.02–12.06). As for PTEN, PTEN-others accounted for 76.6% (131/171) responders, whereas 64% (115/179) were PTEN-loss, with a trend towards lower odds of CR (OR 1.64, 95% CI 0.99–2.74).

PR positivity therefore conferred the largest effect (OR 7.73, 95% CI 2.77–21.63; 5 studies; 199 patients), followed by NSMP subtype (OR 2.04, 95% CI 1.33–3.11; 14 studies; 819 patients). POLEmut, ER positivity, and PTEN-loss were not significantly associated with better CR (OR 1.39, 95% CI 0.60–3.24; OR 1.43, 95% CI 0.52–3.93; OR 1.64; 95% CI 0.99–2.74, respectively). KRASmut (OR 1.80, 95% CI 0.34–9.50) and PIK3CA mutation (OR 0.45, 95% CI 0.02–12.06) estimates were imprecise owing to sparse data.

### 3.2. Secondary Outcomes: Cumulative CR Incidence, Recurrence, Progression, and Reproductive Outcomes

**Figure 3:**
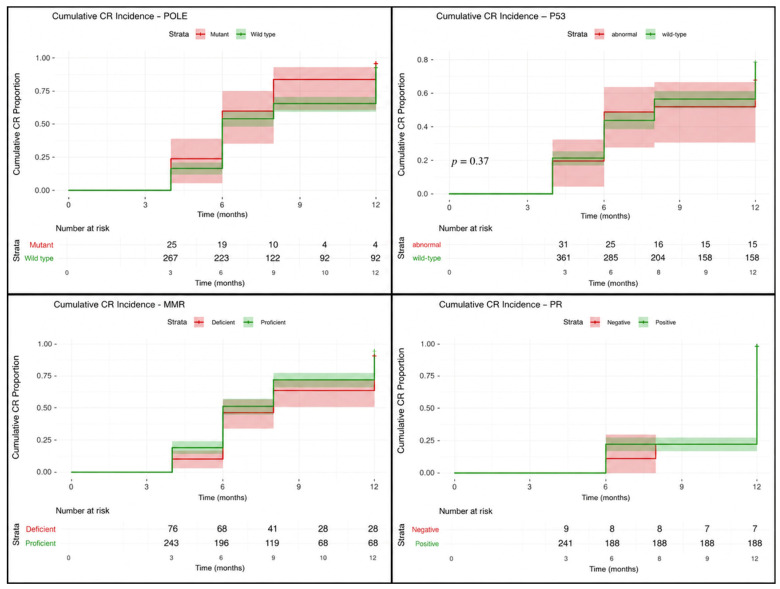
Kaplan–Meier curves for cumulative complete remission incidence by molecular or hormonal profile.

Across all molecular subgroups, the cumulative incidence of CR increased between 4 and 8 months predominantly. Pooled TTR was estimable for biomarkers with adequate reporting. pMMR tumours achieved CR in a pooled median of 7.6 months (95% CI, 7.2–8.1; 9 studies) compared with 6.7 months (95% CI, 6.1–7.3; 10 studies) for dMMR tumours. POLEmut tumours demonstrated the shortest pooled TTR, with 25% of patients achieving CR at 3.9 months (95% CI, 3.2–4.6; 8 studies), compared with 7.0 months (95% CI, 6.5–7.4) for POLE wild-type tumours. PR-positive tumours achieved CR in a median of 12 months (95% CI, 5.8–7.4; 3 studies). Reconstructed cumulative incidence curves of CR were generated for MMR, POLE, p53, and PR status (Figure 3), and the response rates are summarized in Table 2.

**Figure 4:**
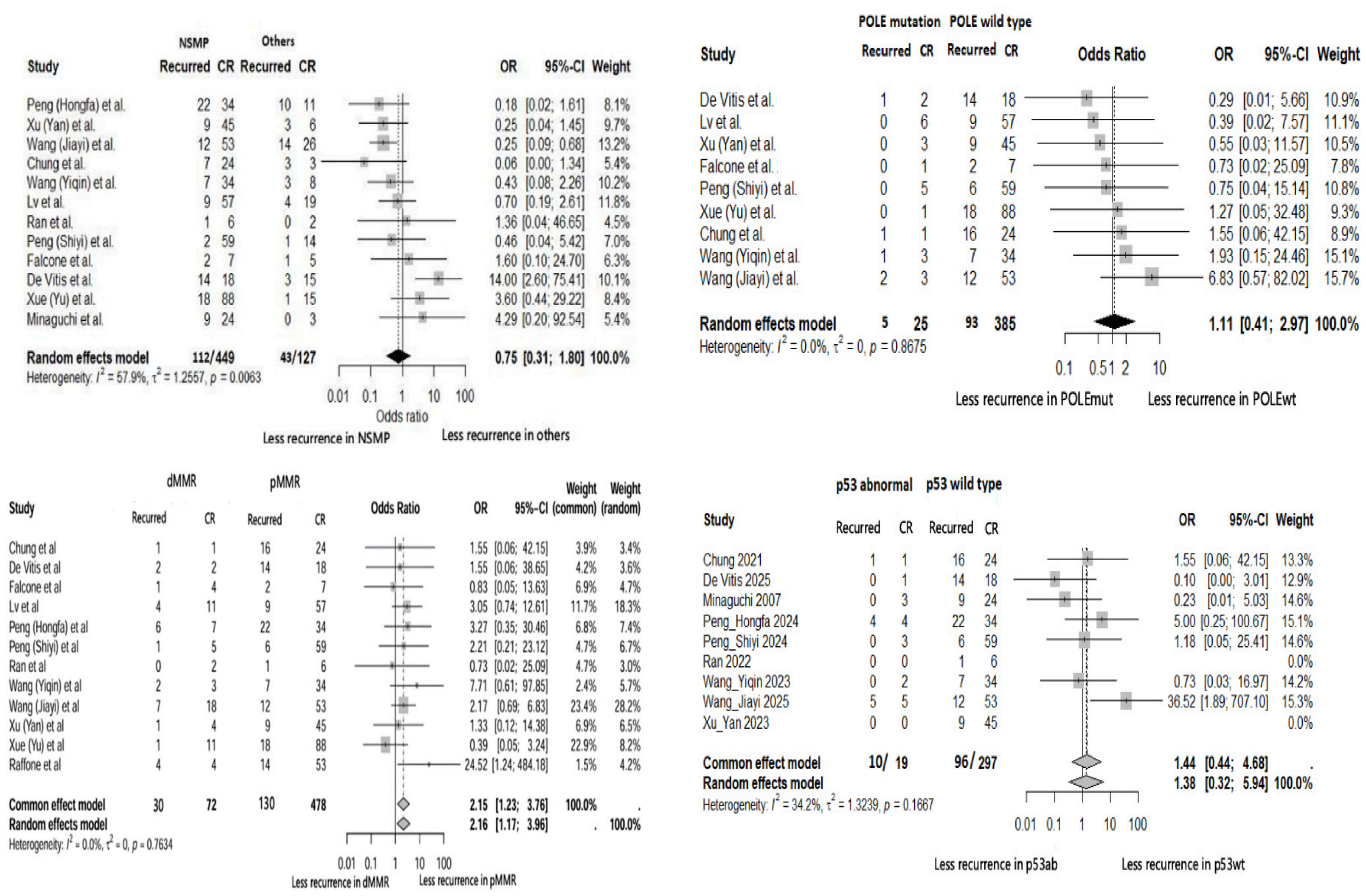
Forest plots for recurrence by molecular classification [30,31,33,34,36,38,40,41,42,43,44,45,46]. Abbreviations: CI: confidence interval; CR: complete remission; NSMP: no specific molecular profile; OR: odds ratio.

After achieving initial CR, we tracked each group to detect recurrence rates. There was not a significant difference in recurrence rates neither with NSMP subtype (OR 0.75, 95% CI 0.31–1.8; 12 studies; I^2^ = 57.9%), nor with the POLEmut status (OR 1.11, 95% CI 0.41–2.97; 9 studies; I^2^ = 0%) or p53abn (OR 1.4, 95% CI 0.32–5.94; 9 studies; I^2^ = 34.2%). Across included studies, dMMR was the only biomarker associated with a significantly increased risk of recurrence (OR 2.15, 95% CI 1.23–3.76; 12 studies; I^2^ = 0%), with no evidence of between-study heterogeneity (Figure 4).

When evaluating the risk of progression, molecular profiling demonstrated profound stratification. Notably, POLEmut tumours were at high risk of progression (OR 0.17, 95% CI 0.06–0.49). Significantly higher risk was also identified for p53abn tumours (OR 0.23, 95% CI 0.08–0.67) and dMMR tumours (OR 0.22, 95% CI 0.11–0.44). NSMP subtype had a significantly lower risk of progression compared to other profiles (OR 0.29, 95% CI 0.16–0.53). As for PR or PTEN statuses, there were no significant differences (OR 0.17, 95% CI 0.01–2.01 and OR 0.58, 95% CI 0.16–2.09, respectively). These results are presented in Supplementary Materials as forest plots.

Reproductive outcomes were inconsistently reported. Assisted reproductive technologies (ART) were frequently used. Overall, reproductive outcomes after fertility-sparing management were highly heterogeneous, with variable reporting of pregnancy and live birth rates, which limited direct comparison across studies. In the study by Wang et al., NSMP tumours showed the most favourable reproductive outcomes, with pregnancy rates reaching 57.7%, and live birth rates nearing 54% following CR. In the same study, dMMR tumours demonstrated 35% pregnancy rates with 60% live birth rates [30]. Another study by Peng et al. demonstrated similar reproductive outcomes for NSMP and dMMR tumours [44]. In both studies, pregnancy rates in patients with POLEmut and p53abn tumours were extremely low due to unspecified reasons.

Meta-regression showed no significant effect of publication year or gene type on CR or progression. In contrast, molecular labelling was a strong moderator, with favourable labels (e.g., positive, wild type) consistently associated with higher CR rates and lower progression risk. Binary marker status confirmed significantly improved CR in marker-positive groups. PR-positivity was associated with increased CR. No molecular factors significantly influenced recurrence, although detection methods incorporating PCR/Sanger sequencing were associated with reduced recurrence compared with immunohistochemistry alone.

## 4. Discussion

In this meta-analysis, significant differences in complete remission, progression, recurrence, and cumulative complete remission incidence were observed according to molecular subtype and hormonal receptor expression, suggesting that these biomarkers may influence response to fertility-sparing treatment. These findings are consistent with those reported by Ferrari et al. [49], who also demonstrated the prognostic value of molecular classification in conservatively managed endometrial lesions.

The primary aim of this systematic review was to contribute to the transition from a “one-treatment-fits-all” approach toward biologically tailored conservative management.

Among molecular classifiers, p53abn tumours consistently demonstrated the poorest outcomes, with lower probabilities of complete remission and markedly increased risks of progression. These findings are consistent with the recognized aggressive genomic instability and potential progesterone-resistant biology associated with this subtype. Collectively, these data support the hypothesis that p53-abnormal status may represent a relative contraindication to prolonged conservative management [50]. Similarly, dMMR tumours exhibited reduced complete remission rates and substantial recurrence and progression risk, reflecting the unclear endocrine responsiveness of this subtype, and suggesting that these tumours may require closer surveillance, careful counselling and maybe more aggressive treatment [7].

In contrast, NSMP tumours demonstrated more favourable outcomes with high remission rates and moderate recurrence risk, supporting their suitability for conservative management as a potentially hormonally driven disease.

Although POLE-mutated tumours are consistently associated with an excellent prognosis in surgically treated patients [51], these favourable outcomes could result from the ultramutated phenotype, which generates a high neoantigen load and a highly immunogenic tumour microenvironment, leading to effective immune surveillance and durable tumour control [52]. Consequently, treatment de-escalation, including omission of adjuvant radiotherapy in selected patients, is increasingly recommended for this molecular subgroup. However, these favourable outcomes reflect prognosis following definitive tumour removal and cannot be directly extrapolated to fertility-sparing management, where long-term disease control depends on hormonal responsiveness. In our meta-analysis, POLE-mutated tumours demonstrated acceptable CR rates and an early response; however, a possible progression risk was observed. This finding should be interpreted with considerable caution, as it is based on a very small number of POLE-mutated cases and sparse progression events, resulting in unstable estimates. Therefore, these observations should be regarded as hypothesis-generating and require confirmation in larger prospective studies with longer follow-up.

As expected, PR-positive status had a major impact on the likelihood of CR, while PR-negative status was linked to a higher risk of recurrence, which highlights the importance of progesterone sensitivity in mediating response to fertility-sparing management. In contrast, ER positivity was associated with only modest improvement in initial response and did not appear to predict durable remission, suggesting that ER expression alone may have limited prognostic value.

However, hormonal receptor expression should be interpreted in conjunction with molecular subtype. Because most tumours were hormone receptor positive, overlap across molecular subgroups was substantial, indicating that hormonal receptor status alone is insufficient to predict treatment response and should not constitute the sole criterion for fertility-sparing management decisions. Further studies evaluating hormonal receptor expression within individual molecular subtypes are required to better define endocrine responsiveness and improve prediction of treatment outcomes.

The presence of somatic mutations was associated with lower odds of reaching CR. In particular, PTEN and PIK3CA mutations had a trend towards less CR, suggesting reduced sensitivity to hormonal therapy. These findings should be interpreted cautiously given the limited available data because we had a very limited sample size.

Time-to-remission analyses indicated that most patients achieved cumulative CR incidence within 6–7 months, with variability across subgroups. These findings emphasize the importance of interpreting treatment duration within a biological context. Premature discontinuation of fertility-sparing treatment in slower responders may unnecessarily limit conservative opportunities, whereas persistent disease in biologically high-risk subgroups may warrant earlier treatment escalation or definitive surgery.

Regarding recurrence after CR, our findings suggest that molecular subgroups including NSMP, POLEmut, and p53abn were not associated with a significant risk of recurrence. In contrast, dMMR was the only biomarker associated with an increased recurrence risk, suggesting that this subgroup may require closer surveillance and further investigation to better understand the underlying mechanisms of relapse. The last finding is consistent with the previous meta-analysis by Zhang et al. [53], which evaluated fertility-sparing treatment outcomes in patients with MSI-H/dMMR AH or EC. Their results similarly suggested that MMR deficiency may represent a subgroup with a higher likelihood of recurrence, highlighting the importance of careful surveillance and individualized management in these patients.

Although evaluation of the efficacy of hysteroscopic resection was not a predefined objective of this meta-analysis, favourable outcomes were reported in several included studies where hysteroscopic resection was combined with progestin therapy, including Falcone et al., Wang J. et al. and Xu et al. [30,31,42]. However, as these studies were not designed to compare treatment modalities, the independent contribution of hysteroscopic resection to treatment response cannot be determined.

According to the ESGO 2023 guidelines, hysteroscopic resection may be considered to achieve optimal cytoreduction and enhance the therapeutic effect of progestin-based treatment [11]. In this context, combining hysteroscopic resection with hormonal therapy may be particularly relevant in biologically unfavourable subgroups demonstrating reduced responsiveness to progestins, such as p53abn or dMMR tumours. However, this hypothesis remains speculative and could be a recommendation for future research.

To our knowledge, the present meta-analysis included the largest number of studies assessing the impact of molecular classification on oncologic outcomes in patients undergoing fertility-sparing treatment for atypical hyperplasia or endometrial cancer. Its strengths lie in a rigorous methodology, including systematic study identification, careful assessment of data quality, and data synthesis. Unlike recent systematic reviews and meta-analyses, which either focused on specific molecular subgroups such as dMMR/MSI tumours [53] or evaluated fertility-sparing treatment modalities, reproductive and oncological outcomes without molecular stratification [54,55], our study provides a comprehensive evaluation of all major molecular classes, including POLEmut, dMMR, p53abn, NSMP and other biomarkers. This molecularly integrated approach allows a more detailed assessment of the prognostic relevance of molecular classification and its potential implications for fertility-sparing management.

Nonetheless, several limitations must be acknowledged. The included studies were exclusively retrospective and involved relatively small sample sizes, particularly for the POLEmut and p53abn subgroups. Although randomized controlled trials represent the highest level of evidence, we did not identify any that evaluated oncologic outcomes according to molecular classification. Most randomized trials in the fertility-sparing setting have focused on comparing treatment strategies rather than assessing outcomes stratified by molecular subtype. Consequently, the available evidence on the prognostic value of molecular classification is currently limited to retrospective observational studies.

A potential limitation of our analysis is the risk of molecular misclassification due to incomplete implementation of the hierarchical molecular classification. In Falcone et al., two tumours showed both dMMR on immunohistochemistry and POLE mutations; after individual review, one case was reclassified as POLE-mutated, whereas the second was retained as dMMR due to a pathogenic germline MSH6 variant [42]. In addition, two studies (Ran et al. and Lv et al.) applied the sequential ProMisE algorithm, in which POLE sequencing is not routinely performed in dMMR tumours [41,45]. Therefore, the presence of POLE mutations coexisting with dMMR cannot be completely excluded, although this limitation affected a small proportion of cases (3/13 and 15/93 dMMR patients, respectively). Future studies using complete molecular profiling will be required to confirm these findings. This highlights the limitation of the ProMisE framework when incompletely applied, and supports the need for integrated molecular profiling, including POLE, MMR, and p53 assessment.

Beyond pooled statistical associations, we sought to translate the above findings into a clinically applicable management framework. Following the diagnosis of AH or early-stage EC, we think that molecular classification combined with ER/PR assessment may allow stratification of patients into three biologically distinct groups: favourable profile (NSMP with positive PR), intermediate profile (POLE-mutated tumours or NSMP with heterogeneous PR expression), and unfavourable profile (p53abn, dMMR, or PR-negative tumours). Importantly, the objective of this proposed classification is not solely to identify patients likely to respond, but also to understand the clinical significance of persistent disease in line with the tumour biology. For example, persistence beyond six months in p53abn disease may not carry the same prognostic implications as persistence in NSMP tumours. Accordingly, we propose in Table 3 a biologically informed management guide intended to assist clinicians in counselling, surveillance planning, and interpretation of treatment response during fertility-sparing management. This framework should be considered hypothesis-generating and requires prospective validation before routine clinical implementation.

## 5. Conclusions

In this meta-analysis, dMMR and p53-abnormal tumours were associated with lower complete remission and higher risk of progression after fertility-sparing treatment, suggesting that molecularly aggressive subtypes may be less sensitive to fertility-sparing management. NSMP showed favourable oncological outcomes by reaching more complete remission and less risk of progression. POLEmut showed no significant differences in CR and had a high risk of progression if CR was not achieved. These findings support the integration of molecular classification into patient selection; however, they should be interpreted cautiously given the incomplete molecular characterization and potential for misclassification in some included studies. Although PR expression was associated with more favourable outcomes, it should not be considered a standalone prognostic marker. Ultimately, tumour biology remains a key determinant of reproductive outcomes, as more aggressive disease may reduce the opportunity to achieve pregnancy before recurrence or treatment failure.

## Figures and Tables

**Table 2:** Overall response rates by biomolecular markers and hormonal receptors.

	POLEmut	dMMR/MSI-H	p53abn	NSMP/CNL	PR Negative	PTEN-Loss	KRAS Mutation
Complete remission	25/33 (75%)	72/116 (62%)	21/37 (56%)	490/640 (76%)	8/19 (42%)	115/179 (64%)	6/15 (40%)
Progression	6/28 (21%)	20/89 (22%)	7/29 (24%)	33/375 (9%)	1/5 (20%)	0/11	NA
Recurrence	5/25 (20%)	30/72 (28%)	10/19 (53%)	112/449 (28%)	NA	10/36 (28%)	NA
TTR (median in months)	6.0	6.7	6.0	7.7	Not reached	8.7	6.0

**Table 3:** Proposed framework for fertility-sparing management of atypical hyperplasia and endometrial cancer based on molecular profile.

Molecular Profile	Suggested Treatment Strategy	Observed Response	Clinical Interpretation
NSMP	Progestin therapy; hysteroscopic resection may be combined in case of suspicious lesions.	CR generally observed within 6–9 months.	If response is slow or partial, treatment continuation with close surveillance (every 3–6 months) may be considered until CR is achieved.
dMMR	Progestin therapy with hysteroscopic resection.	CR may be delayed and is not consistently achieved within 6–9 months.	Delayed CR may indicate reduced treatment sensitivity and risk of treatment failure or disease progression; close surveillance and reassessment of treatment strategy should be considered. Higher risk of recurrence after CR.
p53abn	Progestin therapy with hysteroscopic resection.	Limited evidence suggests a lower likelihood of response and potential for delayed or absent CR.	Delayed CR may suggest treatment resistance and should prompt early reassessment of the treatment strategy by considering radical treatment.
POLEmut	Progestin therapy; hysteroscopic resection may be combined in case of suspicious lesions.	Early CR appears to be more frequently observed, although evidence is limited.	Failure to achieve an early response may indicate treatment failure; close surveillance after CR is recommended given the limited evidence available.

## Data Availability

All data analyzed during this study are included in the published article. More detailed data could be provided upon request.

## Supplementary Materials

The following supporting information can be downloaded at: https://www.mdpi.com/article/10.3390/cancers18152490/s1.

## Abbreviations

The following abbreviations are used in this manuscript:

**Abbreviation**	**Definition**
AH	Atypical Hyperplasia
ART	Assisted Reproductive Technology
BMI	Body Mass Index
CNL	Copy Number Low
CR	Complete Remission
dMMR	Deficient Mismatch Repair
EC	Endometrial Cancer
ER	Estrogen Receptor
FIGO	International Federation of Gynecology and Obstetrics
HR	Hormonal Receptor
IHC	Immunohistochemistry
KRASmut	KRAS-Mutated
KRASwt	KRAS Wild-Type
MSI-H	Microsatellite Instability-High
NA	Not Available
NGS	Next-Generation Sequencing
NOS	Newcastle–Ottawa Scale
NSMP	No Specific Molecular Profile
OR	Odds Ratio
p53abn	p53-Abnormal
p53wt	p53 Wild-Type
PCR	Polymerase Chain Reaction
pMMR	Proficient Mismatch Repair
POLEmut	POLE-Mutated
POLEwt	POLE Wild-Type
PR	Progesterone Receptor
PRISMA	Preferred Reporting Items for Systematic Reviews and Meta-Analyses
ProMisE	Proactive Molecular Risk Classifier for Endometrial Cancer
REML	Restricted Maximum Likelihood
TCGA	The Cancer Genome Atlas
TTR	Time to Remission
