# Supplementary material for: Fertility-Sparing Management of Atypical Hyperplasia and Endometrial Cancer from the Perspective of Molecular and Hormonal Profiles: A Meta-Analysis-Driven Framework
Source: Cancers (Basel). 2026 Aug 4;18(15):2490. doi: 10.3390/cancers18152490 (PMC13464347; doi:10.3390/cancers18152490)
Supplement: Supplementary file 1 [file cancers-18-02490-s001.zip › Forest plots for progression/Supplementary_Forest_MMR_CR_progression.pdf]

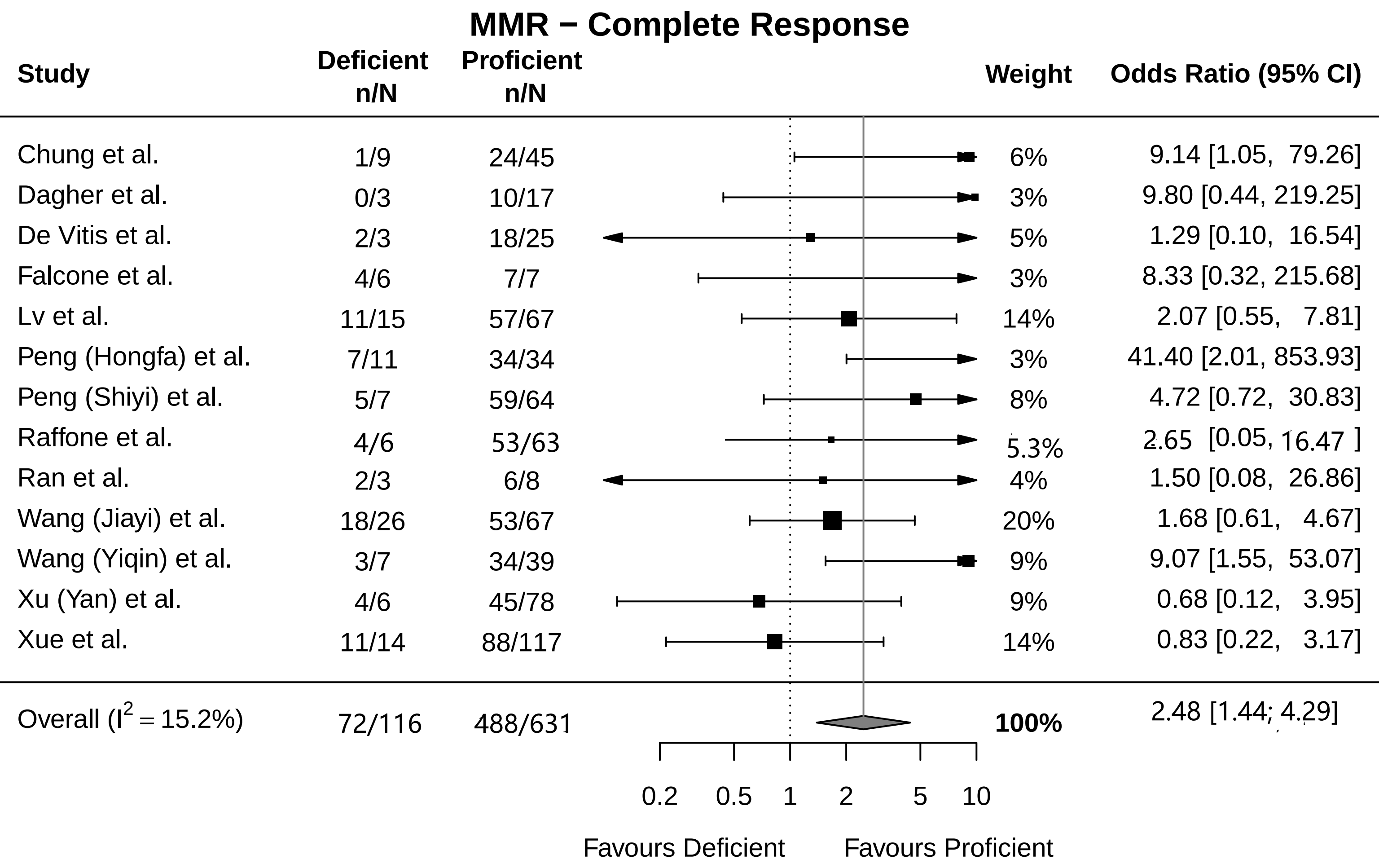
MMR − Complete Response
Study
Deficient n/N
Proficient n/N
Weight
Odds Ratio (95% CI)
Chung et al. 1/9 24/45 6% 9.14 [1.05, 79.26]
Dagher et al. 0/3 10/17 3% 9.80 [0.44, 219.25]
De Vitis et al. 2/3 18/25 5% 1.29 [0.10, 16.54]
Falcone et al. 4/6 7/7 3% 8.33 [0.32, 215.68]
Lv et al. 11/15 57/67 14% 2.07 [0.55, 7.81]
Peng (Hongfa) et al. 7/11 34/34 3% 41.40 [2.01, 853.93]
Peng (Shiyi) et al. 5/7 59/64 8% 4.72 [0.72, 30.83]
Raffone et al. 4/6 53/63 5.3% 2.65 [0.05, 16.47]
Ran et al. 2/3 6/8 4% 1.50 [0.08, 26.86]
Wang (Jiayi) et al. 18/26 53/67 20% 1.68 [0.61, 4.67]
Wang (Yiqin) et al. 3/7 34/39 9% 9.07 [1.55, 53.07]
Xu (Yan) et al. 4/6 45/78 9% 0.68 [0.12, 3.95]
Xue et al. 11/14 88/117 14% 0.83 [0.22, 3.17]
Overall ($I^2 = 15.2\%$) 72/116 488/631 100% 2.48 [1.44; 4.29]
0.2 0.5 1 2 5 10
Favours Deficient
Favours Proficient

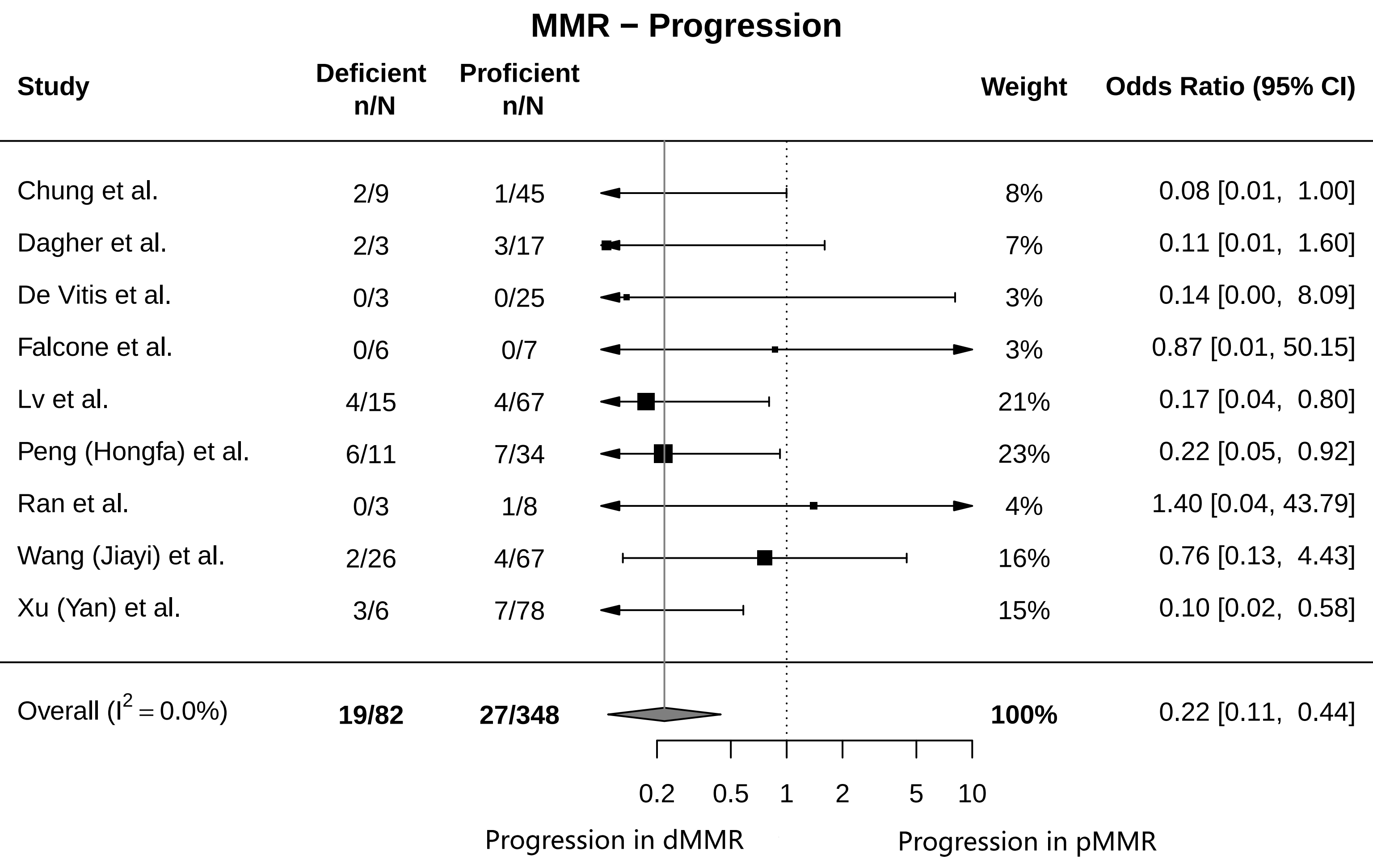
MMR − Progression
Study
Deficient n/N
Proficient n/N
Weight
Odds Ratio (95% CI)
Chung et al. 2/9 1/45 8% 0.08 [0.01, 1.00]
Dagher et al. 2/3 3/17 7% 0.11 [0.01, 1.60]
De Vitis et al. 0/3 0/25 3% 0.14 [0.00, 8.09]
Falcone et al. 0/6 0/7 3% 0.87 [0.01, 50.15]
Lv et al. 4/15 4/67 21% 0.17 [0.04, 0.80]
Peng (Hongfa) et al. 6/11 7/34 23% 0.22 [0.05, 0.92]
Ran et al. 0/3 1/8 4% 1.40 [0.04, 43.79]
Wang (Jiayi) et al. 2/26 4/67 16% 0.76 [0.13, 4.43]
Xu (Yan) et al. 3/6 7/78 15% 0.10 [0.02, 0.58]
Overall ($I^2 = 0.0\%$) 19/82 27/348 100% 0.22 [0.11, 0.44]
0.2 0.5 1 2 5 10
Progression in dMMR
Progression in pMMR
