# Supplementary material for: Fertility-Sparing Management of Atypical Hyperplasia and Endometrial Cancer from the Perspective of Molecular and Hormonal Profiles: A Meta-Analysis-Driven Framework
Source: Cancers (Basel). 2026 Aug 4;18(15):2490. doi: 10.3390/cancers18152490 (PMC13464347; doi:10.3390/cancers18152490)
Supplement: Supplementary file 1 [file cancers-18-02490-s001.zip › Forest plots for progression/Supplementary_Forest_NSMP_CR_progression.pdf]

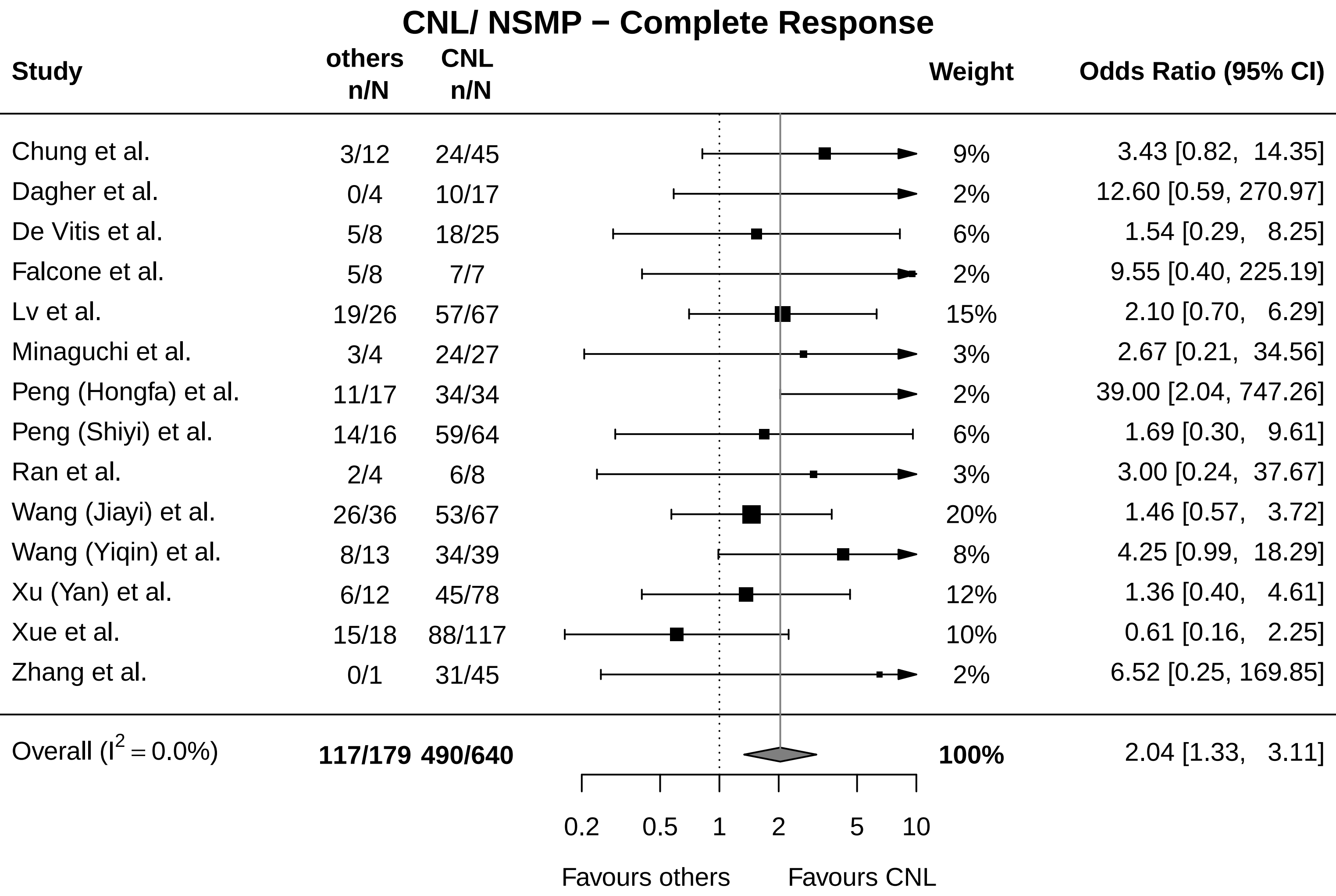
CNL/ NSMP − Complete Response
Study
others n/N
CNL n/N
Weight
Odds Ratio (95% CI)
Chung et al. 3/12 24/45 9% 3.43 [0.82, 14.35]
Dagher et al. 0/4 10/17 2% 12.60 [0.59, 270.97]
De Vitis et al. 5/8 18/25 6% 1.54 [0.29, 8.25]
Falcone et al. 5/8 7/7 2% 9.55 [0.40, 225.19]
Lv et al. 19/26 57/67 15% 2.10 [0.70, 6.29]
Minaguchi et al. 3/4 24/27 3% 2.67 [0.21, 34.56]
Peng (Hongfa) et al. 11/17 34/34 2% 39.00 [2.04, 747.26]
Peng (Shiyi) et al. 14/16 59/64 6% 1.69 [0.30, 9.61]
Ran et al. 2/4 6/8 3% 3.00 [0.24, 37.67]
Wang (Jiayi) et al. 26/36 53/67 20% 1.46 [0.57, 3.72]
Wang (Yiqin) et al. 8/13 34/39 8% 4.25 [0.99, 18.29]
Xu (Yan) et al. 6/12 45/78 12% 1.36 [0.40, 4.61]
Xue et al. 15/18 88/117 10% 0.61 [0.16, 2.25]
Zhang et al. 0/1 31/45 2% 6.52 [0.25, 169.85]
Overall ($I^2 = 0.0\%$) 117/179 490/640 100% 2.04 [1.33, 3.11]
0.2
0.5
1
2
5
10
Favours others
Favours CNL

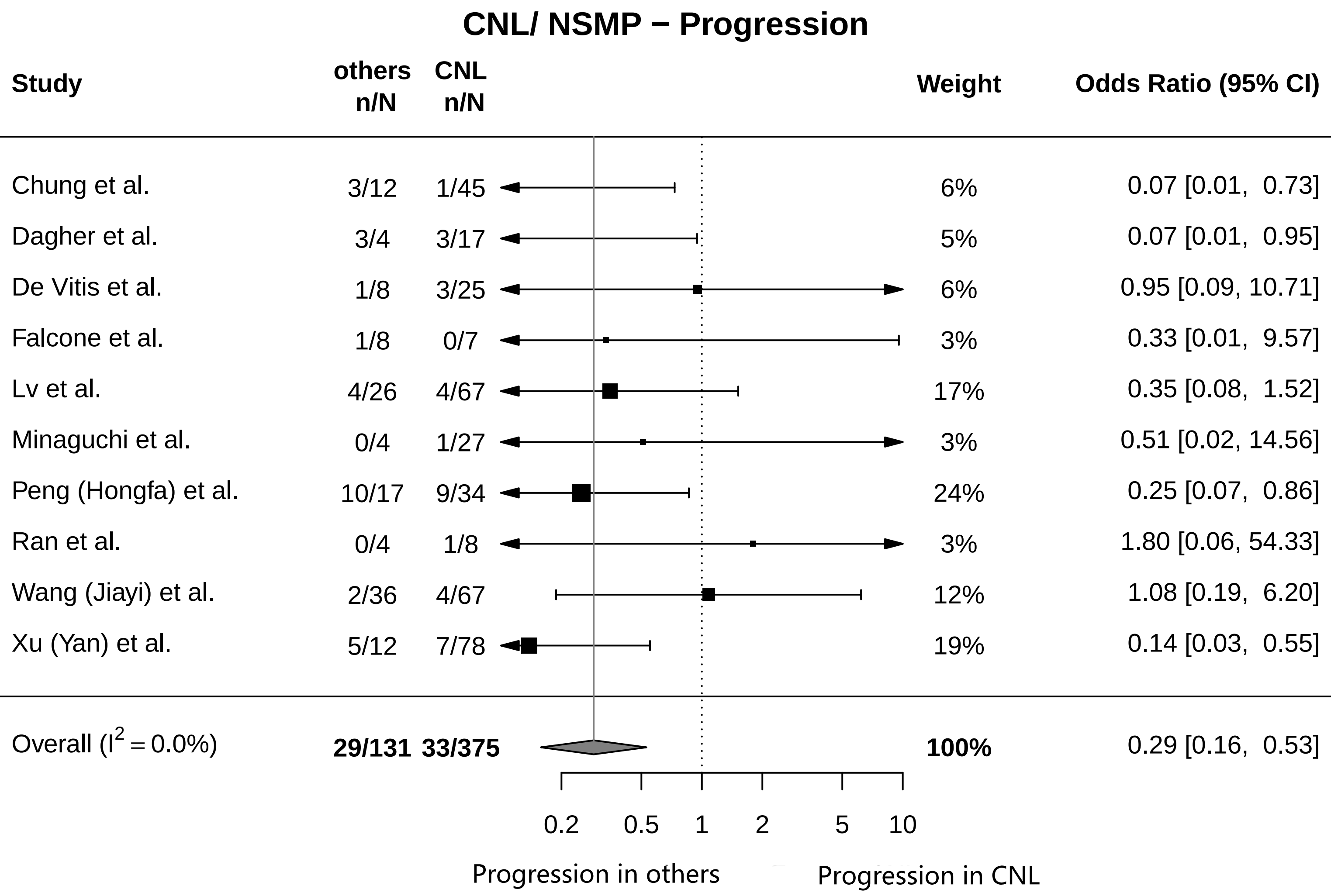
CNL/ NSMP − Progression
Study
others n/N
CNL n/N
Weight
Odds Ratio (95% CI)
Chung et al. 3/12 1/45 6% 0.07 [0.01, 0.73]
Dagher et al. 3/4 3/17 5% 0.07 [0.01, 0.95]
De Vitis et al. 1/8 3/25 6% 0.95 [0.09, 10.71]
Falcone et al. 1/8 0/7 3% 0.33 [0.01, 9.57]
Lv et al. 4/26 4/67 17% 0.35 [0.08, 1.52]
Minaguchi et al. 0/4 1/27 3% 0.51 [0.02, 14.56]
Peng (Hongfa) et al. 10/17 9/34 24% 0.25 [0.07, 0.86]
Ran et al. 0/4 1/8 3% 1.80 [0.06, 54.33]
Wang (Jiayi) et al. 2/36 4/67 12% 1.08 [0.19, 6.20]
Xu (Yan) et al. 5/12 7/78 19% 0.14 [0.03, 0.55]
Overall ($I^2 = 0.0\%$) 29/131 33/375 100% 0.29 [0.16, 0.53]
0.2
0.5
1
2
5
10
Progression in others
Progression in CNL
