# Supplementary material for: Fertility-Sparing Management of Atypical Hyperplasia and Endometrial Cancer from the Perspective of Molecular and Hormonal Profiles: A Meta-Analysis-Driven Framework
Source: Cancers (Basel). 2026 Aug 4;18(15):2490. doi: 10.3390/cancers18152490 (PMC13464347; doi:10.3390/cancers18152490)
Supplement: Supplementary file 1 [file cancers-18-02490-s001.zip › Forest plots for progression/Supplementary_Forest_P53_CR_progression.pdf]

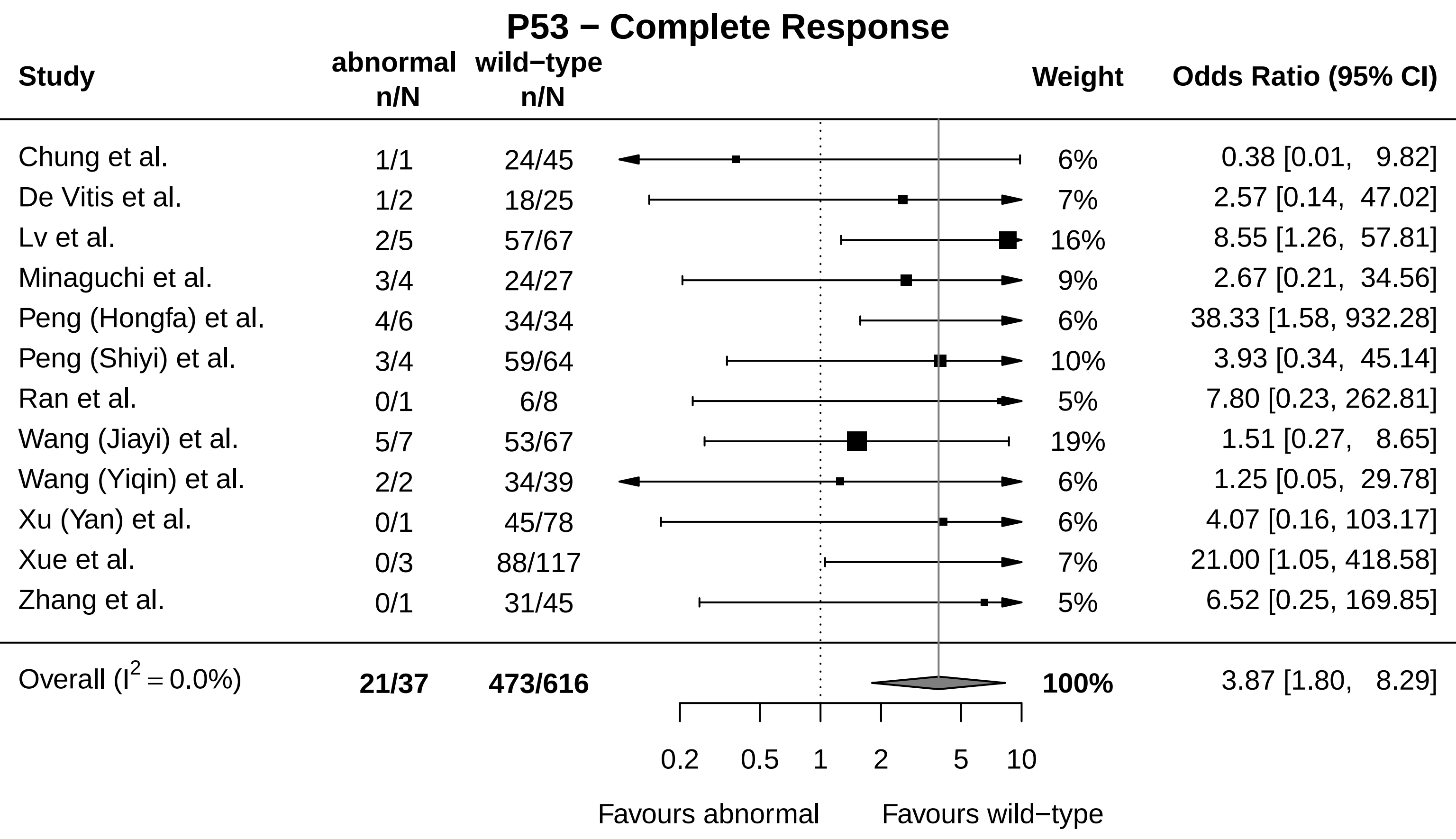
P53 − Complete Response
Study
abnormal n/N
wild−type n/N
Weight
Odds Ratio (95% CI)
Chung et al. 1/1 24/45 6% 0.38 [0.01, 9.82]
De Vitis et al. 1/2 18/25 7% 2.57 [0.14, 47.02]
Lv et al. 2/5 57/67 16% 8.55 [1.26, 57.81]
Minaguchi et al. 3/4 24/27 9% 2.67 [0.21, 34.56]
Peng (Hongfa) et al. 4/6 34/34 6% 38.33 [1.58, 932.28]
Peng (Shiyi) et al. 3/4 59/64 10% 3.93 [0.34, 45.14]
Ran et al. 0/1 6/8 5% 7.80 [0.23, 262.81]
Wang (Jiayi) et al. 5/7 53/67 19% 1.51 [0.27, 8.65]
Wang (Yiqin) et al. 2/2 34/39 6% 1.25 [0.05, 29.78]
Xu (Yan) et al. 0/1 45/78 6% 4.07 [0.16, 103.17]
Xue et al. 0/3 88/117 7% 21.00 [1.05, 418.58]
Zhang et al. 0/1 31/45 5% 6.52 [0.25, 169.85]
Overall ($I^2$ = 0.0%) 21/37 473/616 100% 3.87 [1.80, 8.29]
0.2 0.5 1 2 5 10
Favours abnormal
Favours wild−type

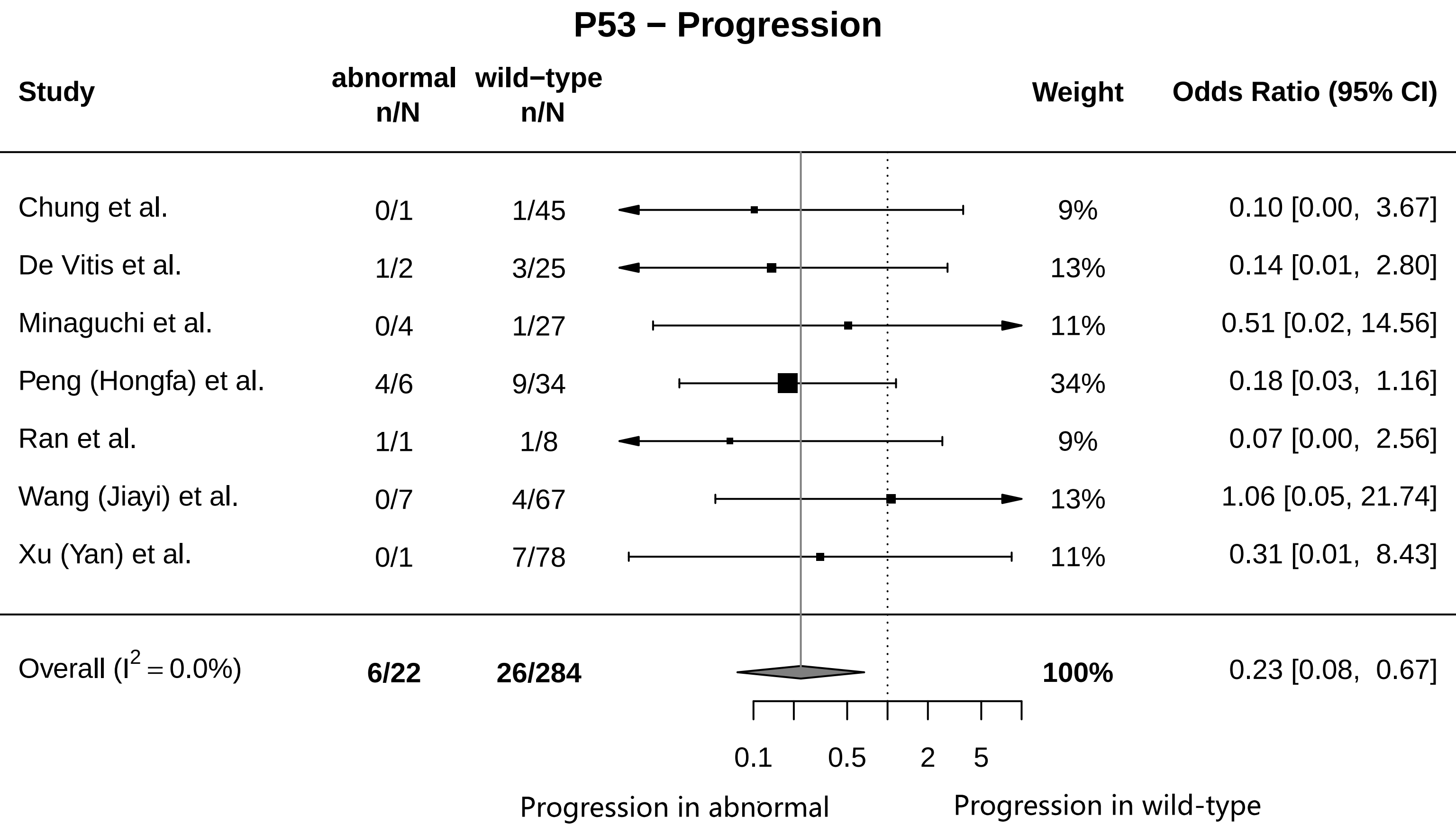
P53 − Progression
Study
abnormal n/N
wild−type n/N
Weight
Odds Ratio (95% CI)
Chung et al. 0/1 1/45 9% 0.10 [0.00, 3.67]
De Vitis et al. 1/2 3/25 13% 0.14 [0.01, 2.80]
Minaguchi et al. 0/4 1/27 11% 0.51 [0.02, 14.56]
Peng (Hongfa) et al. 4/6 9/34 34% 0.18 [0.03, 1.16]
Ran et al. 1/1 1/8 9% 0.07 [0.00, 2.56]
Wang (Jiayi) et al. 0/7 4/67 13% 1.06 [0.05, 21.74]
Xu (Yan) et al. 0/1 7/78 11% 0.31 [0.01, 8.43]
Overall ($I^2$ = 0.0%) 6/22 26/284 100% 0.23 [0.08, 0.67]
0.1 0.5 2 5
Progression in abnormal
Progression in wild-type
