# Supplementary material for: Fertility-Sparing Management of Atypical Hyperplasia and Endometrial Cancer from the Perspective of Molecular and Hormonal Profiles: A Meta-Analysis-Driven Framework
Source: Cancers (Basel). 2026 Aug 4;18(15):2490. doi: 10.3390/cancers18152490 (PMC13464347; doi:10.3390/cancers18152490)
Supplement: Supplementary file 1 [file cancers-18-02490-s001.zip › Forest plots for progression/Supplementary_Forest_POLE_CR_progression.pdf]

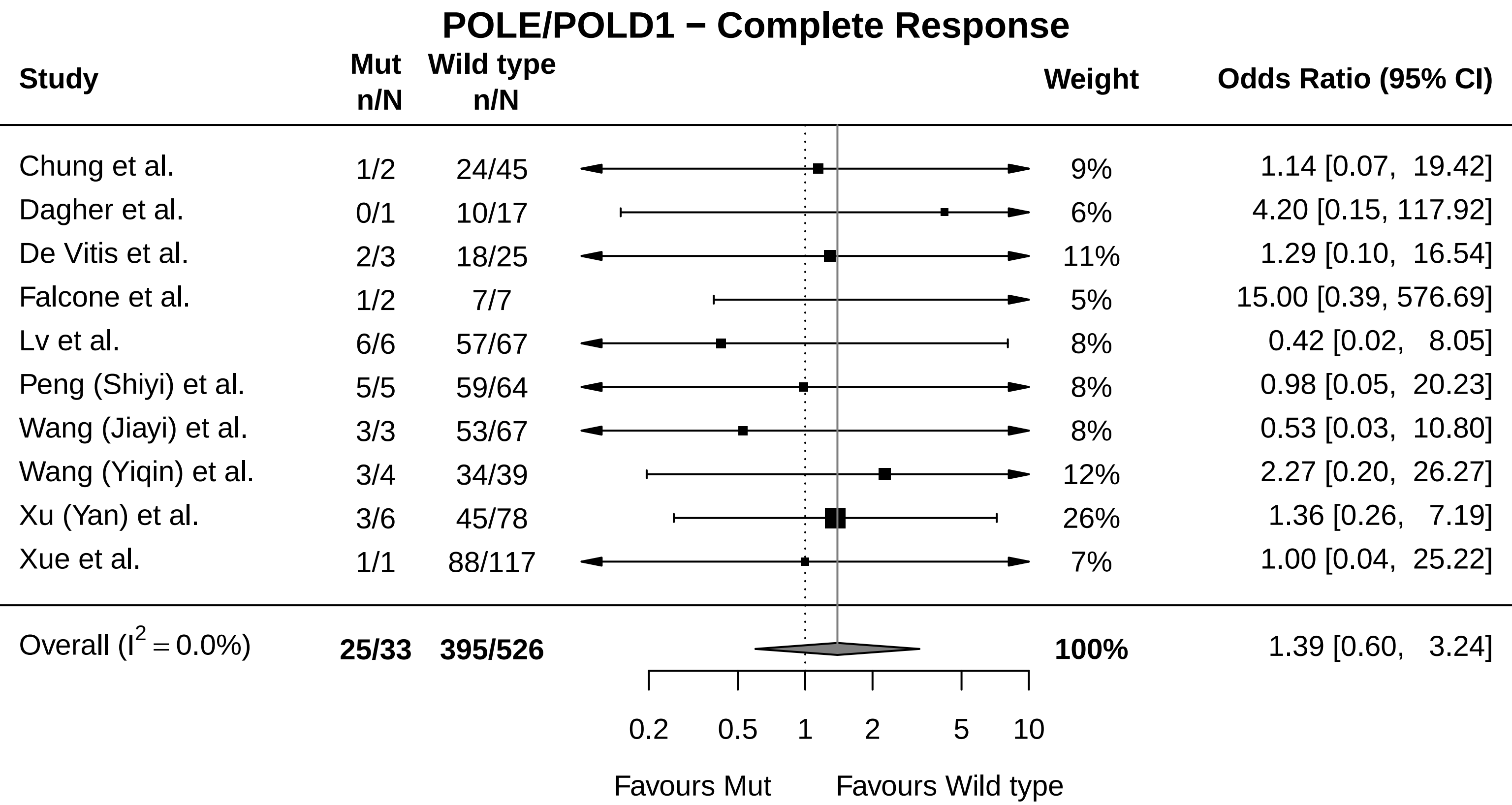
POLE/POLD1 − Complete Response
Study
Mut n/N
Wild type n/N
Weight
Odds Ratio (95% CI)
Chung et al. 1/2 24/45 9% 1.14 [0.07, 19.42]
Dagher et al. 0/1 10/17 6% 4.20 [0.15, 117.92]
De Vitis et al. 2/3 18/25 11% 1.29 [0.10, 16.54]
Falcone et al. 1/2 7/7 5% 15.00 [0.39, 576.69]
Lv et al. 6/6 57/67 8% 0.42 [0.02, 8.05]
Peng (Shiyi) et al. 5/5 59/64 8% 0.98 [0.05, 20.23]
Wang (Jiayi) et al. 3/3 53/67 8% 0.53 [0.03, 10.80]
Wang (Yiqin) et al. 3/4 34/39 12% 2.27 [0.20, 26.27]
Xu (Yan) et al. 3/6 45/78 26% 1.36 [0.26, 7.19]
Xue et al. 1/1 88/117 7% 1.00 [0.04, 25.22]
Overall (I2 = 0.0%) 25/33 395/526 100% 1.39 [0.60, 3.24]
0.2
0.5
1
2
5
10
Favours Mut
Favours Wild type

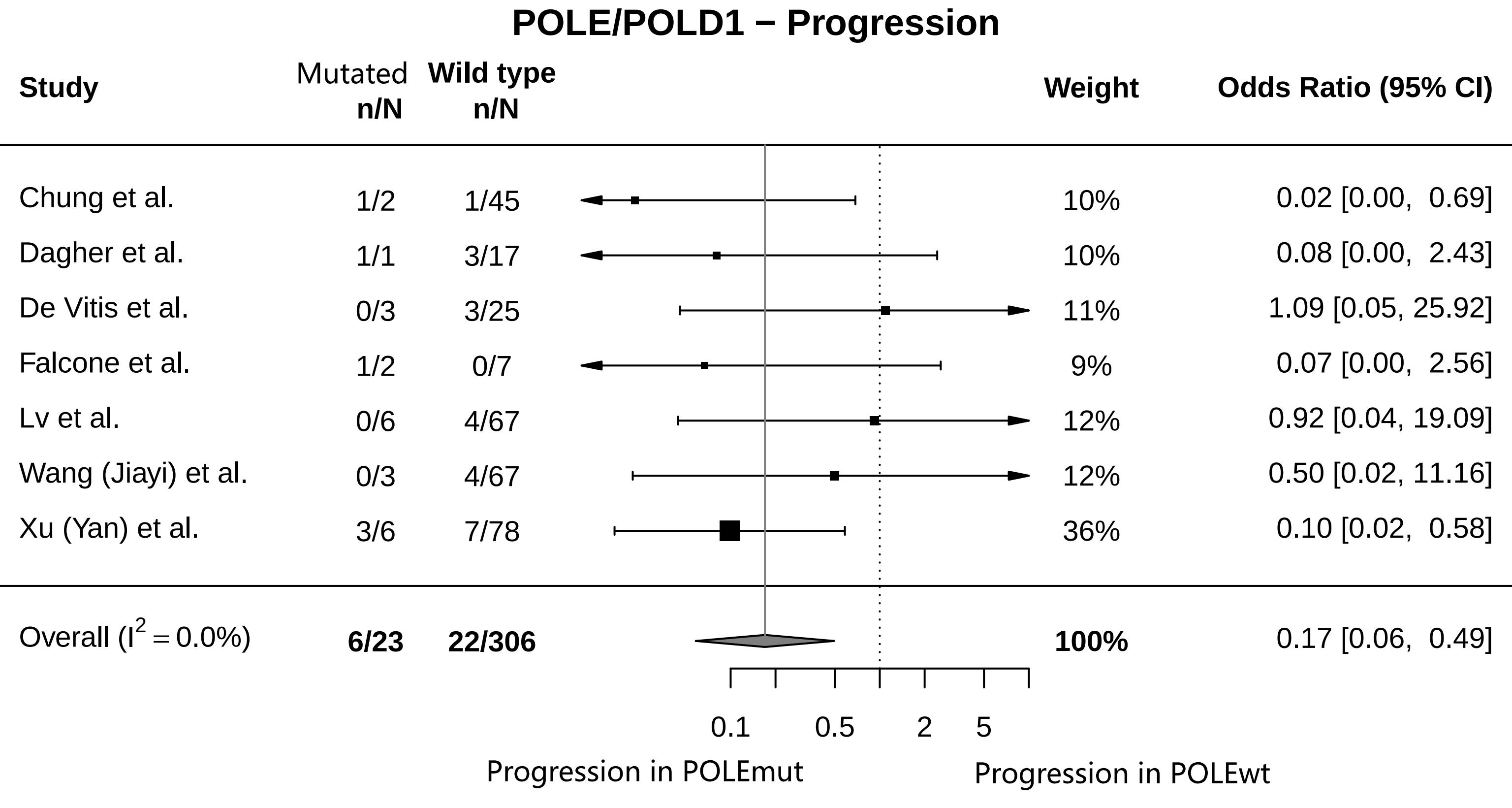
POLE/POLD1 − Progression
Study
Mutated n/N
Wild type n/N
Weight
Odds Ratio (95% CI)
Chung et al. 1/2 1/45 10% 0.02 [0.00, 0.69]
Dagher et al. 1/1 3/17 10% 0.08 [0.00, 2.43]
De Vitis et al. 0/3 3/25 11% 1.09 [0.05, 25.92]
Falcone et al. 1/2 0/7 9% 0.07 [0.00, 2.56]
Lv et al. 0/6 4/67 12% 0.92 [0.04, 19.09]
Wang (Jiayi) et al. 0/3 4/67 12% 0.50 [0.02, 11.16]
Xu (Yan) et al. 3/6 7/78 36% 0.10 [0.02, 0.58]
Overall (I2 = 0.0%) 6/23 22/306 100% 0.17 [0.06, 0.49]
0.1
0.5
2
5
Progression in POLEmut
Progression in POLEwt
