# Supplementary material for: Fertility-Sparing Management of Atypical Hyperplasia and Endometrial Cancer from the Perspective of Molecular and Hormonal Profiles: A Meta-Analysis-Driven Framework
Source: Cancers (Basel). 2026 Aug 4;18(15):2490. doi: 10.3390/cancers18152490 (PMC13464347; doi:10.3390/cancers18152490)
Supplement: Supplementary file 1 [file cancers-18-02490-s001.zip › Forest plots for progression/Supplementary_Forest_PR_CR_progression.pdf]

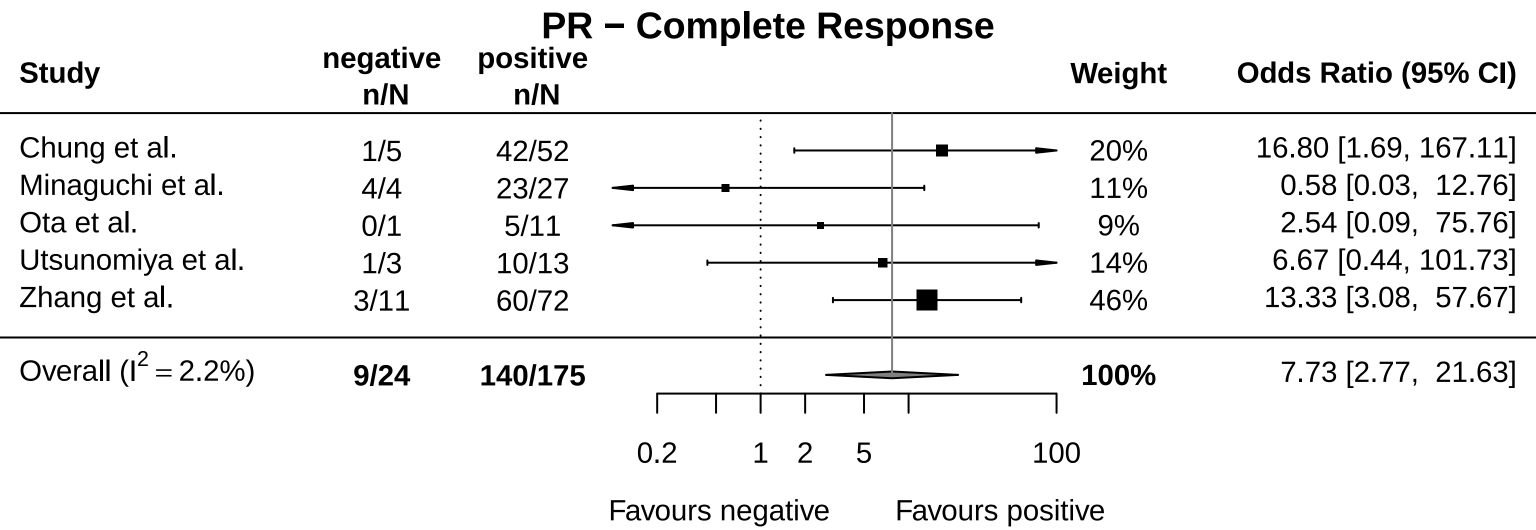
PR − Complete Response
Study
negative n/N
positive n/N
Weight
Odds Ratio (95% CI)
Chung et al. 1/5 42/52 20% 16.80 [1.69, 167.11]
Minaguchi et al. 4/4 23/27 11% 0.58 [0.03, 12.76]
Ota et al. 0/1 5/11 9% 2.54 [0.09, 75.76]
Utsunomiya et al. 1/3 10/13 14% 6.67 [0.44, 101.73]
Zhang et al. 3/11 60/72 46% 13.33 [3.08, 57.67]
Overall (I2 = 2.2%) 9/24 140/175 100% 7.73 [2.77, 21.63]
0.2
1
2
5
100
Favours negative
Favours positive

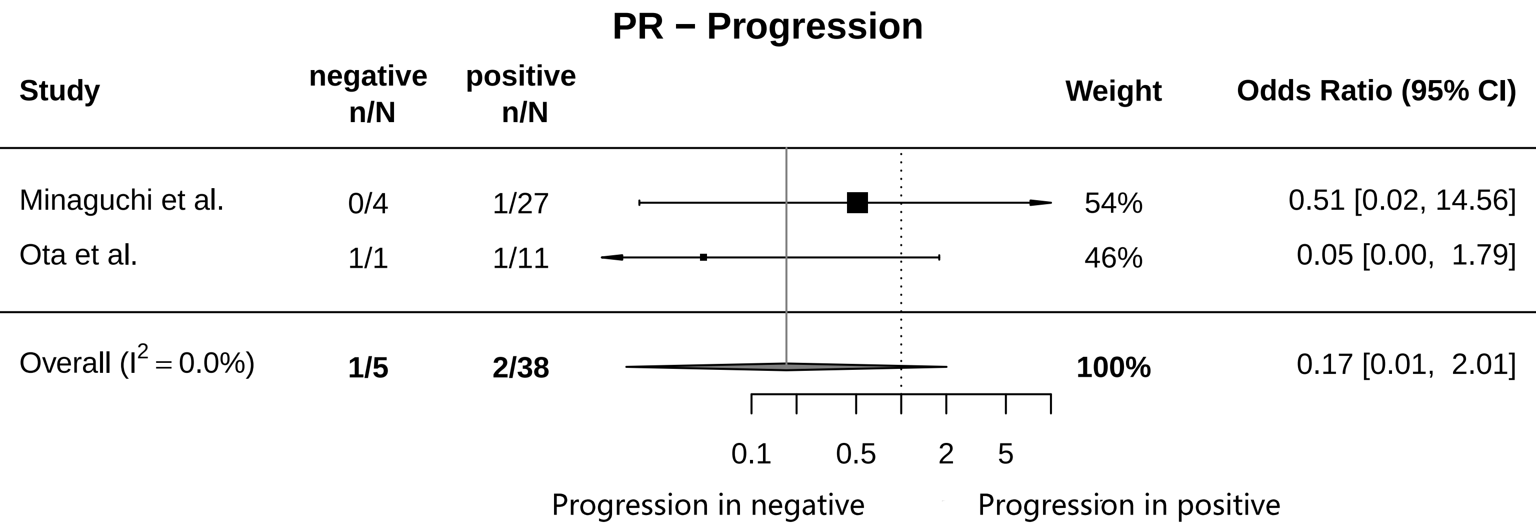
PR − Progression
Study
negative n/N
positive n/N
Weight
Odds Ratio (95% CI)
Minaguchi et al. 0/4 1/27 54% 0.51 [0.02, 14.56]
Ota et al. 1/1 1/11 46% 0.05 [0.00, 1.79]
Overall (I2 = 0.0%) 1/5 2/38 100% 0.17 [0.01, 2.01]
0.1
0.5
2
5
Progression in negative
Progression in positive
