# Supplementary material for: Fertility-Sparing Management of Atypical Hyperplasia and Endometrial Cancer from the Perspective of Molecular and Hormonal Profiles: A Meta-Analysis-Driven Framework
Source: Cancers (Basel). 2026 Aug 4;18(15):2490. doi: 10.3390/cancers18152490 (PMC13464347; doi:10.3390/cancers18152490)
Supplement: Supplementary file 1 [file cancers-18-02490-s001.zip › Forest plots for progression/Supplementary_Forest_PTEN_CR_Progression.pdf]

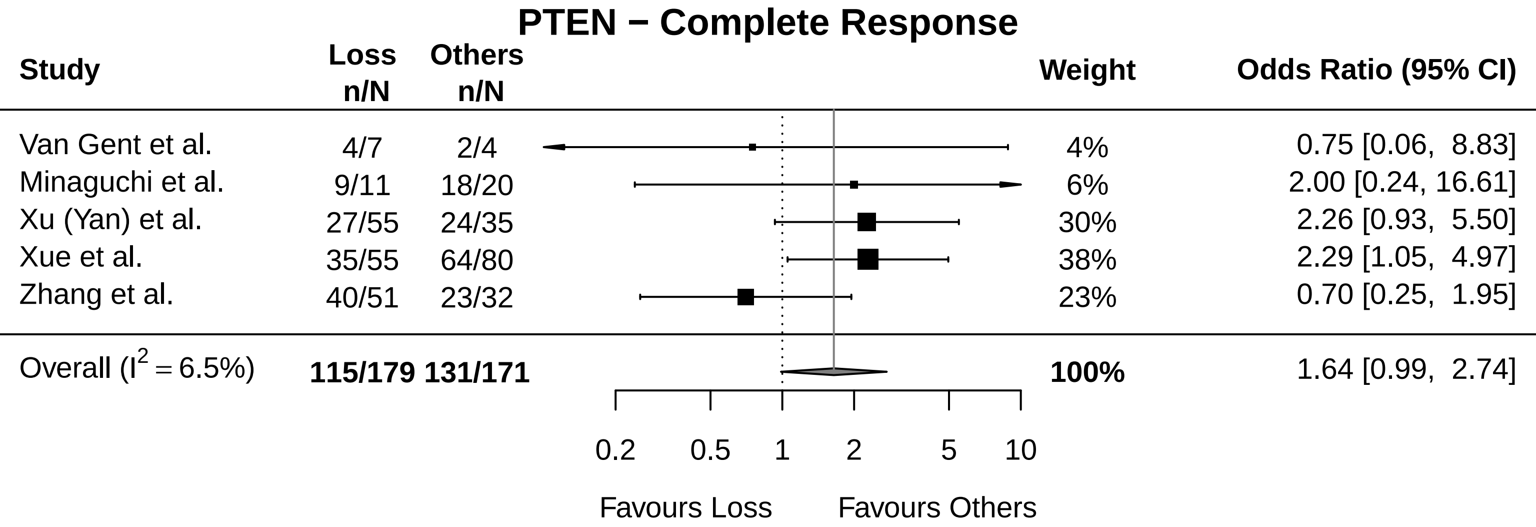
PTEN − Complete Response
Study
Loss n/N
Others n/N
Weight
Odds Ratio (95% CI)
Van Gent et al. 4/7 2/4 4% 0.75 [0.06, 8.83]
Minaguchi et al. 9/11 18/20 6% 2.00 [0.24, 16.61]
Xu (Yan) et al. 27/55 24/35 30% 2.26 [0.93, 5.50]
Xue et al. 35/55 64/80 38% 2.29 [1.05, 4.97]
Zhang et al. 40/51 23/32 23% 0.70 [0.25, 1.95]
Overall ($I^2 = 6.5\%$) 115/179 131/171 100% 1.64 [0.99, 2.74]
0.2
0.5
1
2
5
10
Favours Loss
Favours Others

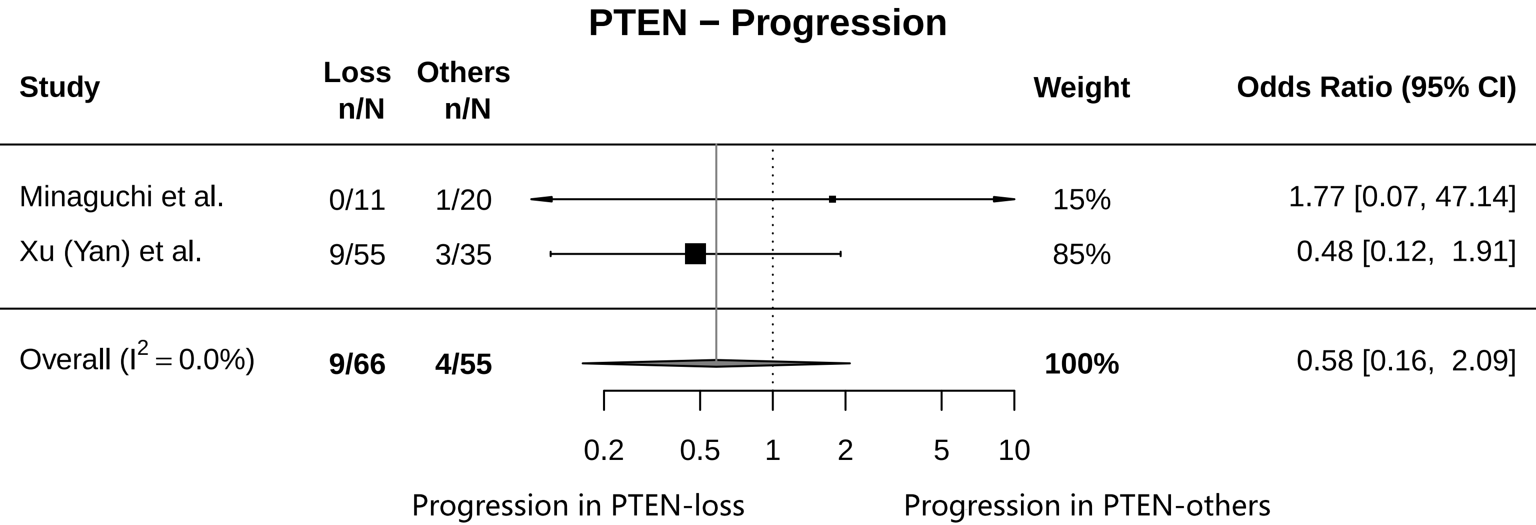
PTEN − Progression
Study
Loss n/N
Others n/N
Weight
Odds Ratio (95% CI)
Minaguchi et al. 0/11 1/20 15% 1.77 [0.07, 47.14]
Xu (Yan) et al. 9/55 3/35 85% 0.48 [0.12, 1.91]
Overall ($I^2 = 0.0\%$) 9/66 4/55 100% 0.58 [0.16, 2.09]
0.2
0.5
1
2
5
10
Progression in PTEN-loss
Progression in PTEN-others
