# Supplementary material for: Fertility-Sparing Management of Atypical Hyperplasia and Endometrial Cancer from the Perspective of Molecular and Hormonal Profiles: A Meta-Analysis-Driven Framework
Source: Cancers (Basel). 2026 Aug 4;18(15):2490. doi: 10.3390/cancers18152490 (PMC13464347; doi:10.3390/cancers18152490)
Supplement: Supplementary file 1 [file cancers-18-02490-s001.zip › Funnel plots/Supplementary_funnel_PR_CR.pdf]

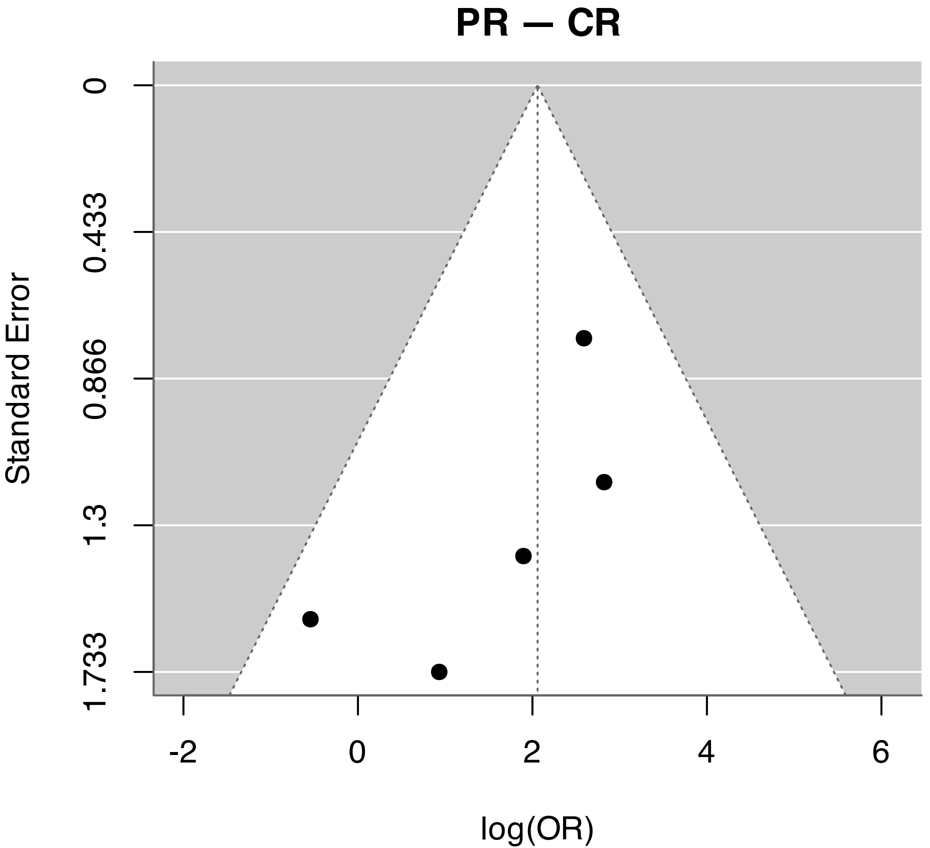

PR — CR
Standard Error
0
0.433
0.866
1.3
1.733
-2
0
2
4
6
log(OR)
